# Predictors of persisting symptoms after concussion in children following a traumatic brain injury: a longitudinal retrospective cohort study

**DOI:** 10.1136/bmjpo-2024-003036

**Published:** 2025-04-05

**Authors:** Rebecca Wilson, Joni Jackson, Kate Birnie, Sharea Ijaz, Matthew Booker, Alex Burrell, Giles Haythornthwaite, Jialan Hong, Mark D Lyttle, Lucy Pocock, Lauren J Scott, Cathy Williams, Ingram Wright, Jelena Savovic, Julie Mytton, Maria Theresa Redaniel

**Affiliations:** 1 NIHR ARC West, Bristol, UK; 2 Population Health Sciences, University of Bristol, Bristol, UK; 3 Centre for Academic Primary Care, University of Bristol Medical School, Bristol, UK; 4 Bristol Royal Hospital for Children Paediatric Emergency Department, Bristol, UK; 5 Research in Emergency Care Avon Collaborative Hub (REACH), University of the West of England, Bristol, UK; 6 University of Bristol School of Clinical Science, Bristol, UK; 7 Centre for Public Health and Wellbeing, University of the West of England, Bristol, UK; 8 National Cancer Registry, Cork, Ireland

**Keywords:** Adolescent Health, Child Health

## Abstract

**Objectives:**

To identify predictors of persisting symptoms after concussion (PSaC) in children, following any medically attended traumatic brain injury (TBI).

**Design:**

Retrospective cohort study.

**Setting:**

Linked primary and secondary care data from UK Clinical Practice Research Datalink and Hospital Episode Statistics.

**Participants:**

Children aged 1–17 years with a medically attended TBI between 2013 and 2017.

**Main outcome measure:**

A binary indicator of PSaC or suspected PSaC, measured using either a clinical code for PSaC or medical attendances for one or more PSaC symptoms 3–12 months after TBI.

**Results:**

We identified 137 873 children with a TBI; 4620 (3.4%) had PSaC or suspected PSaC. More females (3.8%) had PSaC than males (3.1%). Those with PSaC were older at the time of TBI compared with those without PSaC (8 vs 5.5 years). In a multivariable logistic regression model, older age (OR =1.02 per year increase in age, 95% CI 1.01 to 1.03), female sex (OR=1.20, 95% CI 1.13 to 1.28), being Asian (OR=1.37, 95% CI 1.22 to 1.54) or mixed ethnicity (OR=1.18, 95% CI 1.01 to 1.37) (compared with white ethnicity), having a history of headaches (OR=3.52, 95% CI 3.13 to 3.95), learning disabilities (OR=2.06, 95% CI 1.69 to 2.52), ADHD (OR=2.41, 95% CI 1.91 to 3.04), anxiety (OR=2.58, 95% CI 2.18 to 3.05), depression (OR=4.00, 95% CI 3.28 to 4.89) or sleep disorders (OR=2.35, 95% CI 1.99 to 2.78) were associated with increased odds of PSaC.

**Conclusions:**

These results may be used to identify children more likely to develop PSaC following a TBI and those who may benefit from targeted healthcare for PSaC symptoms. Identifying cases of PSaC in primary care data was challenging as perhaps many children do not attend services for suspected PSaC or, if they did, are not diagnosed with PSaC. Furthermore, the clinical predictors are a measure of healthcare access for these symptoms; thus, results could be influenced by patient or carer’s health-seeking behaviour.

**WHAT IS ALREADY KNOWN ON THIS TOPIC**

- Persisting symptoms after concussion (PSaC) is the term used to describe cognitive, physical and psychological symptoms that persist, sometimes for 3 months or more, after a traumatic brain injury (TBI).
- Little is known about why some children recover quickly from a TBI and other children develop PSaC. It is important to identify which children are at risk of PSaC so that clinicians can provide appropriate advice to parents and carers and any interventions for PSaC can be targeted at these children.

**WHAT THIS STUDY ADDS**

- This is the first study to use linked primary and secondary care data from the UK to identify cases of PSaC and suspected PSaC.
- Using linked data, we investigated which demographic and clinical factors were associated with PSaC following a medically attended TBI in 1–17 year olds.
- We found strong evidence that older age, female sex, Asian or mixed ethnicity (compared with white), and histories of headaches, learning disabilities, ADHD, anxiety, depression and sleep disorder were associated with increased risk of PSaC following a medically attended TBI in children.

**HOW THIS STUDY MIGHT AFFECT RESEARCH, PRACTICE OR POLICY**

- Our results may be used to inform care providers which children are more likely to have poorer outcomes following a TBI. They provide the opportunity to offer advice to carers regarding when to seek further care and the potential to target interventions to prevent PSaC"

## Introduction

 Persisting symptoms after concussion (PSaC), previously often referred to as postconcussion syndrome (PCS), describes symptoms causing distress or disability that do not improve, or worsen, after a traumatic brain injury (TBI).^1^ Head injuries, or TBI events, are common in children, with over half a million hospitalisations each year in the UK.^2^ The majority of these TBI events are classified as ‘mild’,^3^ although even mild TBI events can result in poor outcomes and recovery.^4^ Potential symptoms vary and include cognitive, physical and psychological features. Extant literature varies in describing PSaC, including the number of symptoms required to meet a diagnostic threshold for PSaC, or the time after a TBI that symptoms persist.^5^ A widely used evaluation tool, the Rivermead Post Concussion Symptoms Questionnaire,^6^ includes 16 symptoms (headaches, feelings of dizziness, nausea, noise sensitivity, sleep disturbance, fatigue, irritability, feeling depressed, frustration, forgetfulness, poor concentration, taking longer to think, blurred vision, light sensitivity, double vision, and restlessness).

Currently, it is not known why most children make a full recovery within 3 months of TBI, while a small proportion has persistent symptoms. However, intervention at an appropriate timepoint may have a place in ameliorating persistent symptoms.^7 8^ Research into potential predictors of PSaC is growing but is inconsistent. In adults, being female and having a history of ADHD, noise sensitivity, anxiety, depression, bipolar disorder and personality disorder^9^,^11^ are predictors of developing PSaC. A large prospective study of PSaC symptoms in children reported that girls, older children and those with a history of migraine and concussion were at greater risk of PSaC following a TBI,^12^ and further work continues in this area.^13 14^ A recent scoping review^5^ identified 73 publications that examined associations between risk factors and long-term sequelae of TBI in children. The most commonly assessed risk factors were sex, age, prior TBI and injury mechanism. Though results were inconsistent, female sex was associated with worse outcomes; no consistent evidence was found for age, socioeconomic factors, ethnicity, prior TBI, or history of anxiety or depression.

The lack of consistent evidence in this area (which may, in part, be a result of the subjective nature of self-reported symptoms^8^) is likely to mean that children who are at risk of PSaC following a TBI are discharged from care and do not receive appropriate follow-up care or monitoring. Without guidance or indicators of which children are at greater risk of PSaC, clinicians are required to use their judgement about which children to discharge or which parents to offer advice to, which leads to unstandardised care. Knowing which children are at risk of PSaC would support the development of guidance for parents and carers of children with a TBI and could be used to target the most at-risk children if interventions are developed to prevent PSaC. A previous study^12^ developed a clinical risk score for PSaC in children with a TBI using data from an emergency department in Canada. The aim of this study was to identify potential patient predictors of developing PSaC following a TBI in children who attended primary care, visited an accident and emergency department (A&E) or were admitted to hospital.

## Methods

### Study design and data sources

This longitudinal retrospective cohort study used person-level linked data from the Clinical Practice Research Datalink (CPRD) and Hospital Episode Statistics (HES) datasets.

Together, CPRD Aurum^15^ and CPRD Gold^16^ include the electronic primary healthcare records for approximately 60 million patients, including 18 million currently registered, from over 2000 general practices in the UK (https://cprd.com/). CPRD Gold comprises general practitioner’s (GP) practices using Vision software, whereas CPRD Aurum includes those using EMIS software. Secondary care data were obtained via linkage to HES Admitted Patient Care (APC)^17^ and HES A&E data.^18^

Ethical approval for the use of CPRD’s anonymised data is covered by CPRD’s existing ethical approval from the National Research Ethics Service Committee (NRES).^16^ The study protocol is registered on the CPRD database of approved studies (study reference ID: 20_000299). The study Statistical Analysis Plan is provided in the online supplemental material.

The study period was from January 2013 to June 2021 for participants from CPRD Aurum and to August 2021 from CPRD Gold. Linked HES APC and A&E data were available up to October 2020.

### Participants

The study cohort included children who were one to 17 years old between 2013 and 2017 and who visited their GP, visited A&E, or were admitted to hospital following a TBI between 2013 and 2017. The code lists used to identify patients attending with a TBI are available from the code list repository (https://github.com/rebeccawils/PCS-project-code-lists/). The window for capturing TBI attendances was cut-off at 2017, while the study period extended to 2020/21 to allow for up to 4 years’ follow-up data to capture any cases of PSaC or suspected PSaC.

The start of follow-up was the patient registration date in CPRD Aurum or the practice Up to Standard date (the date the GP’s data are considered high enough quality to be used in research) in CPRD Gold. All children were followed up to the earliest of either the patient registration end date (CPRD Aurum only), transfer out date (CPRD Gold only), date of death or practice last collection date at the end of the study period.

### Code list development

Using primary and secondary care data requires the use of code lists to identify consultation codes that indicate the presence of a symptom or health condition or state. CPRD medical codes were used to identify relevant consultations in CPRD Gold and Aurum and ICD 10 codes were used in HES data. We followed the algorithm described by Watson and colleagues.^19^ A member of the research team (JJ, RW or JH) first developed a list of possible codes for a symptom using the CPRD code browser or the ICD code online search tool (https://icd.who.int/browse10/2019/en). This list was provided to two clinicians (JM, MB, AB, MDL, LP, CW or IW) who independently screened each code and labelled it as ‘include’, ‘exclude’ or ‘uncertain’, depending on the likelihood of the code being used in a consultation for that symptom. Codes were retained in the list if one or both clinicians indicated they should be included and were excluded if both clinicians indicated it should be excluded or if one clinician was uncertain and one confident of its exclusion. The finalised code lists were then used to identify any observations, in primary or secondary care data, (ie, healthcare consultations) with the relevant code. The code lists used in the study can be found here: https://github.com/rebeccawils/PCS-project-code-lists/.

### Outcomes

The primary outcome measure was a binary indicator of PSaC defined as a PSaC or PCS diagnosis code recorded at any time (up to the end of the study period) following the TBI event, or one or more PSaC symptom codes recorded between 3 and 12 months after the TBI event (ie, suspected PSaC). The list of PSaC symptoms (headache, dizziness, nausea, noise sensitivity, sleep disturbances, fatigue, feeling irritable or frustrated, depression, forgetfulness, poor concentration, taking longer to think, blurred vision, light sensitivity, double vision and restlessness) was informed by the Rivermead Post Concussion Symptoms Questionnaire^6^ with the addition of disruptive behaviour, as suggested by clinical team members.

As there is no standard definition of PSaC in routinely collected health datasets, we explored the impact of using alternative definitions of the outcome using linked datasets. To explore the impact of changing the threshold of the number of PSaC symptoms and the timeframe in which consultations for PSaC symptoms were captured, three additional binary outcomes were measured: (1) a PSaC or PCS diagnosis code or *two or more* PSaC symptom codes recorded between 3 and 12 months after the TBI; (2) a PSaC or PCS diagnosis code or *one or more* PSaC symptom codes recorded between *3 months and 3 years* after the TBI; and (3) a PSaC or PCS diagnosis code or *two or more* PSaC symptom codes recorded between *3 months and 3 years* after the TBI. Where a PSaC or PCS diagnosis code was used, providing this code was recorded on or after the time of the TBI event, no date restrictions were applied. A minimum of 3 months post-TBI for capturing PSaC or PCS diagnosis was used in line with previous research^20 21^

As three of the PSaC symptoms used to identify suspected PSaC (headaches, depression and sleep disorders) were also investigated as potential predictors of PSaC, we generated an outcome measure for additional sensitivity analysis, which was the same as the primary outcome measure but excluded codes for these three PSaC symptoms.

### Potential predictors

Patient sex and age were obtained from CPRD data and ethnicity and patient area-level deprivation (index of multiple deprivation in lower-layer super output areas (in England^22^) from HES data. Code lists were developed to identify primary and secondary care consultations for the following potential predictors: headaches, learning disabilities, ADHD, anxiety, depression and sleep disorders. For each predictor, a binary variable was generated to indicate if a code was present prior to the date of TBI.

### Statistical analysis

Descriptive analysis was used to explore any differences in age, sex, ethnicity, deprivation and clinical potential predictors between children with and without an indicator for PSaC (ie, those who fulfilled our definition of PSaC or suspected PSaC). Multivariable logistic regression was used to test for associations between all predictor variables and the outcome. Descriptive and regression analyses were performed for the primary outcome and the alternative definitions of the outcome in sensitivity analyses.

We tested for interactions between both age and sex and each of the potential clinical predictors. Where likelihood-ratio tests showed that an interaction term improved the model fit, interactions were explored using the *margins* and *marginsplot* commands^23 24^ in Stata 17.^25^ To investigate how the inclusion of each interaction term affected the multivariable model, we included each single interaction term in a multivariable model and ran a model including all interaction terms, comparing effect sizes across all models.

For the primary outcome variable, we ran least absolute shrinkage and selection operator (LASSO), elastic-net and ridge regression models. We also performed multivariable logistic regression using backwards variable selection at p=0.1. Calibration plots and C-statistics for the full model and backwards selection model were produced. Bootstrapping methods, using 50 samples, were used to test the internal validity of the final model and identify model over-fitting using the *bsvalidation* Stata command.^26^ We obtained the C-statistic and calibration plot from the model produced and compared it with the final model. We conducted a sensitivity analysis for the primary outcome variable model, stratifying the data by age (<5 and ≥5 years).

All analyses were performed using Stata 17.^25^

### Patient and public involvement

Patient and public involvement (PPI) activities have taken place throughout this project. The scoping review that preceded this project^5^ consulted two PPI contributors (one young person with PSaC and one parent of a young person with PSaC) with the preliminary results of the review, and they discussed the list of predictors of PSaC and outcomes (measurements of PSaC) identified in the review. This discussion added patients’ perspectives to the review findings, and these findings informed the development of the list of potential predictors of PSaC and helped refine the outcome measurement used in the current study. The results of the current study were later shared with two young people with a history of PSaC, both due to youth sports. Their opinions on the results and insight into young people’s experience of PSaC and its management helped with our interpretation of the study results.

## Results

We identified 137 873 children with a medically attended TBI between 2013 and 2017. Of these, 4620 (3.4%) had evidence of our primary outcome: a clinical code for PSaC or PCS or suspected PSaC (one or more symptoms between 3 and 12 months after the TBI event). Descriptive statistics are presented in table 1. Numbers of PSaC cases or suspected cases fell to 627 (0.5%) and 2171 (1.6%) when the measure of suspected PSaC was made more stringent/specific (two or more symptoms between 3 and 12 months after the TBI event/two or more symptoms between 3 months and 3 years after the TBI event) and increased to 11 499 (8.3%) when a more sensitive measure of suspected PSaC was employed (one or more PSaC symptoms 3 months to 3 years after the TBI). Descriptive statistics and sensitivity analyses for these alternative definitions of the outcome are presented in the online supplemental tables S1–S5.

**Table 1:** Sample descriptives for the full sample and by primary outcome (a PSaC or PCS diagnosis code or one or more PSaC symptoms 3–12 months after TBI)

	PSaC or suspected PSaC, N (%)			
	No	Yes	Total	
N	133 253 (96.6)	4620 (3.4)	137 873	
Age (years) at time of TBI, mean (SD)	6.47 (5.45)	7.96 (6.53)	6.52 (5.50)	<0.001
Sex				
Male	83 161 (62.4%)	2649 (57.3%)	85 810 (62.2%)	<0.001
Female	50 088 (37.6%)	1971 (42.7%)	52 059 (37.8%)	
Indeterminate*	–(<0.1%)	–(<0.1%)	–(<0.1%)	
Deprivation quintile				
1 (least deprived)	28 861 (21.7%)	989 (21.4%)	29 850 (21.7%)	0.025
2	25 158 (18.9%)	790 (17.1%)	25 948 (18.8%)	
3	23 749 (17.8%)	840 (18.2%)	24 589 (17.8%)	
4	25 587 (19.2%)	922 (20.0%)	26 509 (19.2%)	
5 (most deprived)	29 794 (22.4%)	1077 (23.3%)	30 871 (22.4%)	
Ethnicity				
Asian	7511 (6.0%)	339 (7.8%)	7850 (6.1%)	<0.001
Black	4529 (3.6%)	177 (4.1%)	4706 (3.6%)	
Mixed	4622 (3.7%)	177 (4.1%)	4799 (3.7%)	
Other	2317 (1.9%)	91 (2.1%)	2408 (1.9%)	
White	102 617 (82.3%)	3523 (80.7%)	106 140 (82.2%)	
Unknown	3095 (2.5%)	61 (1.4%)	3156 (2.4%)	
History of headaches†				
No	130 409 (97.9%)	4097 (88.7%)	134 506 (97.6%)	<0.001
Yes	2844 (2.1%)	523 (11.3%)	3367 (2.4%)	
History of learning disability†				
No	132 090 (99.1%)	4483 (97.0%)	136 573 (99.1%)	<0.001
Yes	1163 (0.9%)	137 (3.0%)	1300 (0.9%)	
History of ADHD†				
No	132 590 (99.5%)	4507 (97.6%)	137 097 (99.4%)	<0.001
Yes	663 (0.5%)	113 (2.4%)	776 (0.6%)	
History of anxiety†				
No	132 001 (99.1%)	4358 (94.3%)	136 359 (98.9%)	<0.001
Yes	1252 (0.9%)	262 (5.7%)	1514 (1.1%)	
History of depression†				
No	132 705 (99.6%)	4397 (95.2%)	137 102 (99.4%)	<0.001
Yes	548 (0.4%)	223 (4.8%)	771 (0.6%)	
History of sleep disorder†				
No	131 607 (98.8%)	4419 (95.6%)	136 026 (98.7%)	<0.001
Yes	1646 (1.2%)	201 (4.4%)	1847 (1.3%)	

* Small cell counts suppressed.

† All measured prior to TBI event.

PCSpostconcussion syndromePSaCpersisting symptoms after concussionTBItraumatic brain injury

In a multivariable logistic regression (table 2), older age (OR=1.02, 95% CI 1.01 to 1.03), female sex (OR=1.20, 95% CI 1.13 to 1.28), Asian (OR=1.37, 95% CI 1.22 to 1.54) or mixed (OR=1.18, 95% CI 1.01 to 1.37) ethnicity (compared with white), and history of headaches (OR=3.52, 95% CI 3.13 to 3.95), learning disability (OR=2.06, 95% CI 1.69 to 2.52), ADHD (OR=2.41, 95% CI 1.91 to 3.04), anxiety (OR=2.58, 95% CI 1.91 to 3.04), depression (OR=4.00, 95% CI 3.28 to 4.89) and sleep disorders (OR=2.35, 95% CI 1.99 to 2.78) before the TBI were all associated with increased likelihood of PSaC after a TBI. Deprivation and black and other ethnic groups (compared with the white group) were not associated with PSaC. Multivariable regression models for sensitivity analyses using alternative outcome measures are presented in online supplemental table S5 and stratifying by age in online supplemental table S6.

**Table 2:** Multivariable logistic regression model including primary outcome measure of PSaC or suspected PSaC (PSaC or PCS diagnosis code or one or more PSaC symptoms 3–12 months after TBI)

N=128 966		95% CI	P value
	OR		
Age (years) at time of TBI	1.02	(1.01 to 1.03)	<0.001
Sex (compared with male)			
Female	1.20	(1.13 to 1.28)	<0.001
Deprivation quintile (compared with 1=least deprived)			
2	0.95	(0.86 to 1.05)	0.345
3	1.08	(0.98 to 1.19)	0.144
4	1.06	(0.96 to 1.17)	0.232
5 (most deprived)	1.07	(0.98 to 1.18)	0.141
Ethnicity (compared with white)			
Asian	1.37	(1.22 to 1.54)	<0.001
Black	1.11	(0.95 to 1.30)	0.199
Mixed	1.18	(1.01 to 1.37)	0.041
Other	1.19	(0.96 to 1.48)	0.112
Unknown	0.58	(0.45 to 0.75)	<0.001
History of headaches*	3.52	(3.13 to 3.95)	<0.001
History of learning disability*	2.06	(1.69 to 2.52)	<0.001
History of ADHD*	2.41	(1.91 to 3.04)	<0.001
History of anxiety*	2.58	(2.18 to 3.05)	<0.001
History of depression*	4.00	(3.28 to 4.89)	<0.001
History of sleep disorder*	2.35	(1.99 to 2.78)	<0.001

* Measured before TBI event.

PCSpostconcussion syndromePSaCpersisting symptoms after concussionTBItraumatic brain injury

**Figure 1:**
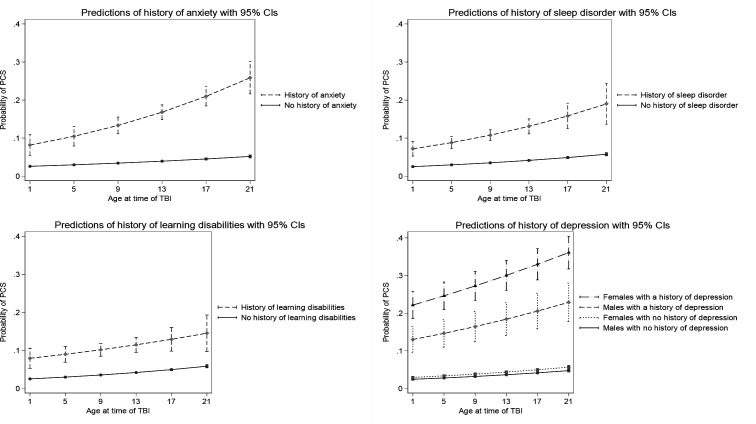
Interactions between: age and anxiety, age and sleep disorders, age and learning disabilities, sex and depression (NB, the ‘Age at time of TBI’ scale goes up to 21 as some participants who were 17 earlier in the study period may have up to 4 years follow-up data). TBI, traumatic brain injury.

Preliminary analysis indicated interactions between having a history of learning disability and age, history of anxiety and age, history of sleep disorder and age, and depression and sex. The margins plots for these interactions are shown in figure 1. These plots indicate that, in unadjusted analyses, the risk of PSaC is greater in children with histories of anxiety, sleep disorder and, to a lesser extent, learning disabilities and that this increased risk is greater in older children, compared with young. Children with a history of depression are also at greater risk of PSaC, and this risk is larger in girls than in boys. However, including each of these interaction terms individually in multivariable models did not alter other effect sizes, and neither did including all of them in one model (online supplemental table S7); therefore, interaction terms were not included in the final model.

There were minimal differences between the full regression model and the model using backwards selection (at p<0.10) (online supplemental table S8). Both models had similar C-statistics (0.6163 and 0.6140) indicating modest model discrimination, and the calibration plots indicate close agreement for predicted and observed values for the primary outcome (online supplemental materials; online supplemental figures S1 and S2). For validation of the full regression model, bootstrapping methods produced an almost identical C-statistic of 0.615, suggesting no model over-fitting. The calibration plot using bootstrapping methods showed slightly less agreement between predicted and observed values for the primary outcome (online supplemental figure S3). The LASSO, elastic net and ridge regression models were almost identical and retained all variables, suggesting good predictive ability (online supplemental table S8).

In sensitivity analyses, the results were generally similar. Using the most stringent outcome (a PSaC or PCS diagnosis code or two or more symptoms 3 months to 1 year after TBI), where just 0.5% of the cohort had the outcome, the associations with sex (OR=1.17, 95% CI 0.99 to 1.39), Asian ethnicity (OR=0.75, 95% CI 0.49 to 1.51), history of learning disability (OR=1.44, 95% CI 0.88 to 2.38), and history of ADHD (OR=1.22, 95% CI 0.70 to 2.11) were attenuated. Evidence remained for associations with age (OR=1.15, 95% CI 1.13 to 1.17), mixed ethnicity (OR=1.51, 95% CI 1.01 to 2.26), and history of headaches (OR=3.57, 95% CI 2.85 to 4.48), anxiety (OR=2.01, 95% CI 1.44 to 2.80), depression (OR=2.39, 95% CI 1.68 to 3.39) and sleep disorder (OR=1.77, 95% CI 1.19 to 2.65). In the model using the most sensitive outcome measure (a PSaC or PCS diagnosis code or one or more symptoms, 3 months to 3 years after TBI), where 8.3% of the cohort had the outcome, in addition to associations observed in the primary outcome model, there was also evidence of an association between the three most deprived quintiles (compared with the least deprived quintile) and increased likelihood of PSaC (quintile 3 OR=1.09, 95% CI 1.02 to 1.16; quintile 4 OR=1.10, 95% CI 1.03 to 1.17; quintile 5 OR=1.14, 95% CI 1.08 to 1.22). Removing the symptoms included in the analysis as potential predictors of PSaC from the symptoms of suspected PSaC (headaches, depression and sleep disorders) produced results that were very similar to the primary outcome model (see online supplemental table S5). This reassured us that the associations observed between clinical histories and the likelihood of PSaC were not explained by these symptoms, which were captured in both the exposures and the outcome.

In further sensitivity analysis stratifying the multivariable logistic regression model with the primary outcome measure (PSaC or PCS diagnosis code or one or more PSaC symptoms 3–12 months after TBI) by age group (under 5 years old and 5 years and older), we observed an inverse association between age and likelihood of PCS in the ≤5-year-olds (OR=0.84, 95% CI 0.82 to 0.87). A positive association remained in the model include the over 5-year-olds (OR=1.08, 95% CI 1.07 to 1.09), indicating that the youngest and the oldest children had the greatest risk of PSaC. The association between female sex and increased risk of PSaC was attenuated in under 5-year-olds (OR=0.97, 95% CI 0.89 to 1.06), and the association between Asian ethnicity and increased risk of PSaC was attenuated in 5 years and older (OR=1.29, 95% CI 1.08 to 1.54). In under 5 years, there was an association between the most deprived quintile and increased likelihood of PSaC (OR=1.18, 95% CI 1.03 to 1.35), compared with the least deprived quintile. In under 5 years, the association between history of ADHD and increased likelihood of PSaC was attenuated, and history of depression was omitted from the model (see online supplemental table S6).

## Discussion

Our analysis showed that older age, being female, being of Asian or mixed ethnicity (compared with white ethnicity), and having a history of headaches, learning disability, ADHD, anxiety, depression and sleep disorders were associated with a greater likelihood of PSaC following a TBI for which children visited primary care or A&E or were admitted to hospital. Our results were consistent across sensitivity analyses where the number and type of PSaC symptoms and the timeframe for medical attendance for symptoms were altered.

Our results suggest that older age is associated with increased likelihood of PSaC, in line with some previous studies.^12 21 27^ However, the literature has shown inconsistent evidence for the association between age and PSaC.^5^ It is difficult to compare results across studies concerning age as other studies used a binary measure of age.^12 27^ We used a continuous measure of age and included a wide age range in our sample. Other studies often focused on a narrower age range, for example, adolescents.^9 28 29^ When stratifying by age group (under 5 and 5 years and older), the results showed that the youngest and oldest children were most at risk of PSaC, further suggesting that the wide age range used in our study is likely to explain these differences and perhaps using a narrower age range is beneficial.

Female sex was a strong predictor of PSaC, consistent with previous evidence.^12 21 27 30^ Reasons for this are unclear. Potential explanations include physiological differences between males and females that result in greater propensity for concussion and its sequelae in girls, greater likelihood of symptom reporting in girls or different mechanisms of injury between sexes.^30^ In sensitivity analysis, the association was attenuated in under 5 years, indicating that it is the older girls (aged 5 years and older) who are at greater risk of PSaC. This may suggest that it is in older childhood when either or both the experiences and physical differences between boys and girls become more disparate. It is also worth noting that, across a wide range of studies that explored sex as a predictor of varied post-concussion outcomes (5), the evidence was, on the whole, mixed.

It was hypothesised that children from lower socioeconomic groups would be more likely to develop PSaC as head injuries occur more frequently in children from the most deprived homes,^31^ and low socioeconomic status was previously shown to be associated with poor outcomes following TBI in children.^32^ We did not find evidence for an association between socioeconomic status and PSaC in the primary analysis. A potential explanation could be that we would expect to see more children with head injuries in lower socioeconomic groups^31 33^ and our sample included only children with a TBI, which would negate the association between socioeconomic status and head injury. We may not have found reliable evidence for an association between socioeconomic status and PSaC due to our measurement of the outcome, which was clinical attendance for PSaC. Clinical attendance for PSaC is likely underreported, as intimated by the low numbers of clinical codes for PSaC or PCS, which suggests our primary outcome is underestimated and impacts the interpretation of our results. Our study might differ from previous research, in that we were essentially measuring health-seeking behaviour in a cohort of patients with a head injury. However, the two sensitivity analyses that included longer follow-up periods for suspected PSaC (up to 3 years) did show associations between the most deprived quintiles and increased risk of PSaC, compared with the least deprived. This may support the hypothesis that social and environmental factors have a larger impact on more long-term recovery following TBI.^34^ Further, when stratified by age group, an association was observed between the most deprived quintile and increased likelihood of PSaC, compared with the least deprived quintile, in the younger children (under 5 years s). It is unclear whether this is due to the nature of the injury or manifestation of symptoms in younger children or the health-seeking behaviour of parents of younger children.

Furthermore, measurement of socioeconomic status differs between studies. We used an area-level measure of patient deprivation, while other measurements of socioeconomic status used previously were often patient-level, including family’s occupation^32^ and health insurance status.^21^ Only in the most sensitive sensitivity analysis (where the outcome included a PSaC or PCS diagnosis code or one or more PSaC symptoms between 3 months and 3 years after a TBI) did we observe associations, in increasing strength, between the three most deprived quintiles and risk of PSaC, compared with the least deprived group. This may suggest that in a larger sample size, or when using a more sensitive measure of PSaC (which might have been the case in previous studies), an association could be observed between socioeconomic status and likelihood of PSaC.

We found some evidence that being Asian or of mixed ethnicity was associated with increased odds of PSaC, compared with white children. Again, it is difficult to know whether this represents an association with health-seeking, rather than incidence of PSaC. These associations were attenuated in the more stringent sensitivity analysis (PSaC or PCS diagnosis code or two or more PSaC symptoms between 3 months and 3 years after a TBI) and in older children when stratifying for age group, suggesting the evidence may be weaker for an association with ethnicity. Previous evidence on ethnicity and PSaC is sparse. One previous study found no evidence of a relationship between ethnicity (white compared with non-white, or non-Hispanic compared with Hispanic) and PSaC.^21^ A study assessing factors associated with length of recovery time in children with PSaC (an outcome that could overlap with ours given that we counted presentations within a time window post TBI) found that white children had a longer recovery time (in median/mean number of days) than Hispanic and African-American children.^35^ Again, comparisons between our results and those published previously are challenging as groupings of ethnicities often vary between studies.

The strongest predictors in our model were the clinical indicators; measures of histories of headaches, learning disability, ADHD, anxiety, depression and sleep disorder that preceded the TBI. While the effect sizes for the associations with clinical predictors were large, as we used primary and secondary care data, we must acknowledge that we are measuring help-seeking and there is the possibility that our results indicate that those who access primary care for one health complaint are more likely to access help for another (ie, PSaC). Previous literature has explored various clinical predictors of PSaC, particularly in adults. The history of ADHD, anxiety and depression was shown to be associated with PSaC in adults,^9 11^ and we have demonstrated this to be evident in children. In children, previous research reported that having ADHD was associated with a longer PSaC recovery period;^35^ however, one study^12^ did not find an association between ADHD and PSaC. The observed association between ADHD and PSaC was attenuated in younger children (under 5 years) in the age group stratified analysis, probably because children of this age are unlikely to receive a diagnosis of ADHD.^36^

The evidence is mixed for psychiatric disorders. While some studies have shown that a history of psychiatric disorder and a history of depression and anxiety were associated with increased odds of PSaC,^12 29 37 38^ one study reported no association between history of anxiety and depression and PSaC.^28^ In line with our results, Zemek and colleagues^12^ also found an association between learning disabilities and history of migraines and increased risk of PSaC. These differences between studies and their results are likely to occur because of measurement variability. Our measures of clinical histories depended entirely on health-seeking and symptom reporting, which has additional challenges in child cohorts, as capturing these data relies on parents, caregivers and healthcare professionals. Further, the measure of history of depression was omitted from the model in younger children when stratifying by age group, probably because of the low number of events (diagnoses of depression) in this age group.

The C-statistic for the full regression model was lower than that reported previously by Zemek *et al*.^12^ There are several possible reasons for this; Zemek and colleagues’ model combined predictors from the derived prediction model with physician judgement regarding the likelihood of recovery, and they used variables generally not available in routinely collected data, whereas our study was likely limited by the available data in linked CPRD datasets.

### Strengths and limitations

Using linked primary and secondary care data allowed us access to a large representative dataset where we could identify all clinically attended TBIs and cases of PSaC and suspected PSaC in England using routinely collected data. Our sample size was relatively large, and we were able to develop robust models that generated consistent and reliable results.

Using routinely collected data also presents limitations, particularly in terms of data availability. Previous TBI, or concussion, is a predictor of PSaC often cited in the literature.^12 35^ However, this was not possible to include in our study as our cohort was identified by their first recorded TBI. Using secondary data meant that we were not able to include other measures that we would have liked to assess, such as information on the injury event that led to the TBI, symptoms present at the time of the TBI or other health-seeking behaviours. Information around the severity of the TBI is not routinely coded in primary care data, and we could not distinguish between mild and severe cases of TBI without imposing subjective judgements by categorising available TBI codes. This does result in a study limitation whereby the inclusion of the entire spectrum of head injuries may affect the results. However, we believed this was the most appropriate way of managing TBI codes as the use of a proxy measure of severity would have brought further limitations.

Measuring PSaC in primary care was a challenge as clinical codes for PSaC or PCS were rarely used and the number of cases was small. Previous research has suggested that PSaC occurs in up to 30% of people with a TBI.^27 39 40^ If this is true, PSaC is clearly underreported in primary and secondary care, or is seldom attended to. To manage this, we attempted to identify suspected cases of PSaC using clinical codes for common symptoms; these lists of symptoms were co-developed with clinical colleagues, including clinicians from psychology, emergency medicine and primary care. However, it is likely that we both missed some cases of PSaC and also miscategorised some children who did not have PSaC as cases. We completed several sensitivity analyses to test different ways of measuring the outcome, which showed consistent results, but there will inevitably be some misclassification.

## Conclusion and implications

Our study produced robust results indicating demographic (patient age, sex and ethnicity) and clinical factors (histories of headaches, learning disability, ADHD, anxiety, depression and sleep disorder) associated with developing PSaC after a TBI. If clinicians know which children are more likely to have poorer outcomes following a TBI, it provides the opportunity to offer advice to carers regarding when to seek further care and the potential to target interventions to prevent PSaC.

## supplementary material

10.1136/bmjpo-2024-003036 online supplemental file 1

10.1136/bmjpo-2024-003036 online supplemental file 2

## Data Availability

Data may be obtained from a third party and are not publicly available.

## References

[1] Broshek DK, Pardini JE, Herring SA (2022). Persisting symptoms after concussion: Time for a paradigm shift. PM R.

[2] National Clinical Guideline Centre (UK) (2014). Head injury: triage, assessment, investigation and early management of head injury in children, young people and adults. https://pubmed.ncbi.nlm.nih.gov/25340248/.

[3] Najmi VS, Yellamraju SK, Toman E (2024). Uncomplicated linear skull fractures in the paediatric population: a retrospective observational study in a UK Major Trauma Centre. Br J Neurosurg.

[4] Sariaslan A, Sharp DJ, D’Onofrio BM (2016). Long-Term Outcomes Associated with Traumatic Brain Injury in Childhood and Adolescence: A Nationwide Swedish Cohort Study of a Wide Range of Medical and Social Outcomes. PLoS Med.

[5] Ijaz S, Scott L, Dawson S (2023). Factors related to adverse long-term outcomes after mild traumatic brain injury in children: a scoping review. Arch Dis Child.

[6] King NS, Crawford S, Wenden FJ (1995). The Rivermead Post Concussion Symptoms Questionnaire: a measure of symptoms commonly experienced after head injury and its reliability. J Neurol.

[7] Ponsford J, Draper K, Schönberger M (2008). Functional outcome 10 years after traumatic brain injury: its relationship with demographic, injury severity, and cognitive and emotional status. J Int Neuropsychol Soc.

[8] Beauchamp MH, Dégeilh F, Rose SC (2023). Improving outcome after paediatric concussion: challenges and possibilities. Lancet Child Adolesc Health.

[9] Houck Z, Asken B, Bauer R (2019). Predictors of post-concussion symptom severity in a university-based concussion clinic. *Brain Inj*.

[10] Dischinger PC, Ryb GE, Kufera JA (2009). Early Predictors of Postconcussive Syndrome in a Population of Trauma Patients With Mild Traumatic Brain Injury. Journal of Trauma.

[11] Langer LK, Alavinia SM, Lawrence DW (2021). Prediction of risk of prolonged post-concussion symptoms: Derivation and validation of the TRICORDRR (Toronto Rehabilitation Institute Concussion Outcome Determination and Rehab Recommendations) score. PLoS Med.

[12] Zemek R, Barrowman N, Freedman SB (2016). Clinical Risk Score for Persistent Postconcussion Symptoms Among Children With Acute Concussion in the ED. *JAMA*.

[13] Bressan S, Takagi M, Anderson V (2016). Protocol for a prospective, longitudinal, cohort study of postconcussive symptoms in children: the Take C.A.Re (Concussion Assessment and Recovery Research) study. BMJ Open.

[14] Yeates KO, Beauchamp M, Craig W (2017). Advancing Concussion Assessment in Pediatrics (A-CAP): a prospective, concurrent cohort, longitudinal study of mild traumatic brain injury in children: protocol study. BMJ Open.

[15] Wolf A, Dedman D, Campbell J (2019). Data resource profile: Clinical Practice Research Datalink (CPRD) Aurum. Int J Epidemiol.

[16] Herrett E, Gallagher AM, Bhaskaran K (2015). Data Resource Profile: Clinical Practice Research Datalink (CPRD). Int J Epidemiol.

[17] Herbert A, Wijlaars L, Zylbersztejn A (2017). Data Resource Profile: Hospital Episode Statistics Admitted Patient Care (HES APC). Int J Epidemiol.

[18] NHS England (2024). Hospital episode statistics (HES). https://digital.nhs.uk/data-and-information/data-tools-and-services/data-services/hospital-episode-statistics.

[19] Watson J, Nicholson BD, Hamilton W (2017). Identifying clinical features in primary care electronic health record studies: methods for codelist development. BMJ Open.

[20] Barlow KM, Crawford S, Brooks BL (2015). The Incidence of Postconcussion Syndrome Remains Stable Following Mild Traumatic Brain Injury in Children. Pediatr Neurol.

[21] Babcock L, Byczkowski T, Wade SL (2013). Predicting postconcussion syndrome after mild traumatic brain injury in children and adolescents who present to the emergency department. JAMA Pediatr.

[22] McLennan D, Barnes H, Noble M (2011). English indices of deprivation 2010: technical report. https://www.gov.uk/government/statistics/english-indices-of-deprivation-2010-technical-report.

[23] Xu J, Long JS (2005). Confidence Intervals for Predicted Outcomes in Regression Models for Categorical Outcomes. *Stata J*.

[24] Long JS (2006). Group comparisons and other issues in interpreting models for categorical outcomes using stata.

[25] StataCorp (2021). Stata statistical software: release 17.

[26] Fernandez-Felix BM, García-Esquinas E, Muriel A (2021). Bootstrap internal validation command for predictive logistic regression models. *Stata J*.

[27] Fried E, Balla U, Catalogna M (2022). Persistent post-concussive syndrome in children after mild traumatic brain injury is prevalent and vastly underdiagnosed. Sci Rep.

[28] Bunt SC, Meredith-Duliba T, Didehhani N (2021). Resilience and recovery from sports related concussion in adolescents and young adults. J Clin Exp Neuropsychol.

[29] Presley C, Meredith-Duliba T, Tarkenton T (2020). A-32 Acute Concussive Symptom Profiles in Adolescents and Young Adults with History of Depression and Anxiety. Arch Clin Neuropsychol.

[30] Bretzin AC, Covassin T, Wiebe DJ (2021). Association of Sex With Adolescent Soccer Concussion Incidence and Characteristics. JAMA Netw Open.

[31] Marlow R, Mytton J, Maconochie IK (2015). Trends in admission and death rates due to paediatric head injury in England, 2000-2011. Arch Dis Child.

[32] Jones KM, Barker-Collo S, Parmar P (2018). Trajectories in health recovery in the 12 months following a mild traumatic brain injury in children: findings from the BIONIC Study. *J Prim Health Care*.

[33] Edwards P, Green J, Lachowycz K (2008). Serious injuries in children: variation by area deprivation and settlement type. Arch Dis Child.

[34] Muscara F, Catroppa C, Eren S (2009). The impact of injury severity on long-term social outcome following paediatric traumatic brain injury. Neuropsychol Rehabil.

[35] Aggarwal SS, Ott SD, Padhye NS (2020). Sex, race, ADHD, and prior concussions as predictors of concussion recovery in adolescents. Brain Inj.

[36] Great Ormond Street Hospital NHS Foundation Trust (2016). Attention deficit hyperactivity disorder (ADHD). https://www.gosh.nhs.uk/conditions-and-treatments/general-medical-conditions/attention-deficit-hyperactivity-disorder-adhd/.

[37] Presley CR, T C, Tarkenton T (2020). A-147 Post-Concussive Symptoms in Adolescent Athletes with Premorbid Psychiatric History: A Matched Case–Control Study. Arch Clin Neuropsychol.

[38] Olsson KA, Lloyd OT, Lebrocque RM (2013). Predictors of child post-concussion symptoms at 6 and 18 months following mild traumatic brain injury. Brain Inj.

[39] McMahon P, Hricik A, Yue JK (2014). Symptomatology and functional outcome in mild traumatic brain injury: results from the prospective TRACK-TBI study. J Neurotrauma.

[40] van der Vlegel M, Polinder S, Toet H (2021). Prevalence of Post-Concussion-Like Symptoms in the General Injury Population and the Association with Health-Related Quality of Life, Health Care Use, and Return to Work. J Clin Med.

