# Supplementary material for: Predictors of persisting symptoms after concussion in children following a traumatic brain injury: a longitudinal retrospective cohort study
Source: BMJ Paediatr Open. 2025 Apr 5;9(1):e003036. doi: 10.1136/bmjpo-2024-003036 (PMC11973773; doi:10.1136/bmjpo-2024-003036)
Supplement: online supplemental file 1 [file bmjpo-9-1-s001.pdf]

NIHR | Collaboration for Leadership in Applied Health Research and Care West

Predictors of post-concussion syndrome in children following a traumatic brain injury

Statistical Analysis Plan

	Name	Title	Signature	Date
**SAP author**	Rebecca Wilson	Senior Research Associate		
**SAP authoriser**	Theresa Redaniel	Senior Lecturer		
**SAP authoriser**	Kate Birnie	Research Fellow		

**Effective Date:**	

Table of Contents

1. Introduction to the SAP 3
1.1 Scope 3
1.2 Document approval 3
1.3 Updates to the document following approval 3
1.4 Skeleton tables and figures 3
2. Abbreviations and Definitions 3
3. background, objectives and outcomes 4
3.1 Background 4
3.2 Aim 4
3.3 Objectives 4
3.4 Primary outcome 4
4. methods 4
4.1 Design and population 4
4.2 Analysis groups 5
4.3 Consent 5
5. Data collection 5
6. Data sources 7
7. Statistical analyses 8
7.1 Descriptive data 8
7.2 Model development 8
7.3 Internal validation of the model 8
7.3.1 Data presentation and analysis models 9
7.3.2 Subgroup analyses 9
7.3.3 Sensitivity analyses 9
7.3.4 Missing data 9
7.4 Statistical software 9
8. References 10
Appendix A: Skeleton tables and figures 11
APPENDIX B: CRPD Codes 14
PCS Codes 14
TBI Codes 15
Migraines and headaches codes ..... **Error! Bookmark not defined.**
Learning disabilities codes ..... **Error! Bookmark not defined.**
ADHD Codes ..... **Error! Bookmark not defined.**
Depression codes ..... **Error! Bookmark not defined.**
Anxiety codes ..... **Error! Bookmark not defined.**
Sleep disorders codes ..... **Error! Bookmark not defined.**

1. INTRODUCTION TO THE SAP

1.1 Scope

This document details information regarding the statistical analysis of a project investigating predictors and outcomes of traumatic brain injury (TBI) in children, specifically the objective investigating predictors of post-concussion syndrome (PCS). The analysis will be conducted by the National Institute for Health Research Applied Research Collaboration West (NIHR ARC West).

1.2 Document approval

The ARC West Applied Data Science Team lead Theresa Redaniel and external statistical consultant Kate Birnie will authorise this document. Other members of the project team will also be invited to comment prior to approval.

1.3 Updates to the document following approval

Any changes made to this statistical analysis plan (SAP) after approval must be clearly justified and documented as an amendment below. The SAP should then be re-approved.

1.4 Skeleton tables and figures

Throughout this document references are made to any skeleton tables and figures to be used in the reporting of the project (e.g. **Table 1** or **Figure 1**). Such tables and figures can be found in **Appendix A** of this document and are intended as a guide for evaluation reporting. Final versions of the tables/figures may differ- tables may be combined, and/or their layout or numbering may change. However, the content should be consistent with the tables and figures in **Appendix A**.

2. ABBREVIATIONS AND DEFINITIONS

**Abbreviations**	**Definitions**
ARC	Applied Research Collaboration
CI	Confidence Interval
CPRD	Clinical Practice Research Datalink
HES	Hospital Episode Statistics
IMD	Index Multiple Deprivation
OR	Odds Ratio
PCS	Post-concussion syndrome
SAP	Statistical Analysis Plan
TBI	Traumatic brain injury

3. BACKGROUND, OBJECTIVES AND OUTCOMES

3.1 Background

Traumatic brain injury (TBI) is the leading cause of death and disability in people aged 1-40 years in the UK (1). Although most of the Emergency Department (ED) attendances have mild TBI or trivial head injury; even mild TBI (also known as concussion) can cause long term adversities for the patient, family, healthcare system, and society. Because children's brains are still developing, even mild TBI may result in intellectual, academic and personality adjustment problems (2).

Following a TBI, some people develop post-concussion syndrome (PCS), which includes somatic, cognitive, psychological, and/or behavioural symptoms that can continue for several months (3), though there is not a consensus on the definition (4). Approximately one third of children who have a concussion will experience PCS up to three months following a TBI (5,6), which can result in unrecognised difficulties with academic performance and social interaction. Whilst most recover over time, a proportion of cases experience longer term physical and psychological symptoms. However, it is likely that early recognition and targeted intervention speed recovery and reduce persistence of symptoms in the longer term.

In order to target interventions at children who are at higher risk of developing PCS, it is important to know who the risk groups are. For children aged 1-17 we are interested in identifying predictors of PCS following any medically attended TBI event.

3.2 Aim

To identify predictors of PCS following a medically attended TBI in children aged 1-17.

3.3 Objectives

1. Identify the predictors of PCS using linked CPRD Gold/Aurum and HES data.
2. Internal validation of the model developed in CPRD Gold/Aurum, using bootstrapping

3.4 Primary outcome

The outcome of interest is PCS (see Appendix B for PCS codes) at least three months after a TBI event and within one year of a TBI event. Cases of PCS were identified using codes for a diagnosis of PCS or a symptom of PCS (see Appendix for codes) within three months and one year of the TBI event. This will be measured using a binary variable (0 = no PCS, 1 = PCS).

4. METHODS

4.1 Design and population

This is a retrospective cohort study, including all patients aged 1-17 years, who were registered at a participating GP practice between 2013-2017, with a TBI event. The start of data accrual for each patient is the date the patient joined the practice or the CPRD 'up to standard' date (for CPRD GOLD only), whichever is later, and the start of follow-up is the TBI event date. The follow up period will be up to one year after the index event (TBI event). The study end is the earliest of: one year post TBI event, the date of patient transfer-out from practice, the date of patient's death (according to ONS or CPRD death date) or the last date of data collection from that practice. (If a patient

had more than one TBI event they would be included more than once, with one follow up period for each TBI event.)

4.2 Analysis groups

Patients (a cohort all with a TBI event between 2013-2017) will be identified in CPRD data using two sets of codes for TBI for CPRD Gold and CPRD Aurum (see Appendix B). There are N=137,279 cases of TBI (using the 'broad definition' of TBI).

4.3 Consent

All data used in this study are routinely collected and anonymous and therefore this does not require patient consent. GPs do not need to seek individual patient consent when they share data with CPRD, however patients can opt out of patient information being shared for research.

4.4 Withdrawals

Withdrawal from the study is not possible as only routine data are being used. However, patients who have opted to have their data removed from data registries will be excluded from analyses as their data will not be available.

5. DATA COLLECTION

Outcome: Post-concussion syndrome – any recorded indicator of PCS (a diagnosis of PCS or at least one PCS symptom) as per the list of codes for CPRD Gold and Aurum and HES data (Appendix B) a minimum of three months after and up to one year after the TBI event for main analysis. Code lists were based on a search of terms from key words and clinical expertise.

Cohort definition: TBI event – all included patients will have a recorded event as per the codes used for CPRD Gold and Aurum (Appendix B), in particular this is a subset of patients whose TBI events are coded as abusive or are reported alongside a code for domestic abuse

Patient characteristics: age, sex, ethnicity, area-level deprivation

Potential predictors (identified from a scoping review conducted by a member of the team and from expert opinion): age at time of TBI, prior concussion, time since last concussion, physician diagnosed history of migraines or headaches, learning disabilities, attention deficit (hyperactive) disorder, anxiety, depression, and sleep disorders, plus any additional factors identified in the scoping review. Not all of these predictors will be available/appropriate for inclusion at all ages (eg, depression is unlikely to be recorded for young children), some predictors will be included only in certain age-stratified models (see table of candidate predictors below). (Codes included in Appendix B) Code lists were developed by initially by the research term, using search terms to search the CPRD (Gold and Aurum) code dictionaries for potentially relevant codes. These code lists were then sent to two clinicians for checking. Clinicians coded codes as either 'definitely include', 'definitely exclude' or 'maybe'. Codes coded as 'definitely include' or 'definitely exclude' by both clinicians were included or excluded, respectively. Where there was uncertainty, either due to 'maybe' codes or discrepancies between clinicians' coding, agreement was met through discussion between clinicians and researchers.

Candidate predictor definitions

Predictor	Definition	Source/code list
Age	Child's age at the time of the TBI in years	CPRD data
Socioeconomic status	Categorical variable indicating Index of Multiple Deprivation (IMD), in quintiles from 1 (least deprived) to 5 (most deprived).<br><br>Patient level IMD as a proxy for socioeconomic status. Where patients do not have patient level IMD we will use practice level IMD.<br><br>IMD is one of the most commonly used measures of deprivation. It is a composite measure derived from of a number of indicators covering different aspects ('domains') of material deprivation: income, employment, education and skills, health, housing, crime, access to services, and living environment. Each domain index can itself be a composite score derived from two or more sub-domain indicators. The overall composite index, the IMD, is calculated as a weighted sum of the domain indices.<br><br>The first official 'Indices of Deprivation' for England were produced by the UK Department for Communities and Local Government in 2000, replacing the 1998 Index of Local Deprivation. Updates for 2004, 2007, 2010 and 2015 were calculated at lower layer super output area (LSOA) level.	Linkage to practice level index of multiple deprivation and the patient-level indices of multiple deprivation (IMD)
Ethnicity	Patient's ethnicity will be obtained from HES APC dataset and categorised as white, Black, Asian, mixed or other.	Linkage with HES Admitted Patient Care dataset
History of concussion	Binary variable indicating previous concussion (before TBI event) recorded in child's medical records, using codes provided in Appendix B	CPRD data
Time since concussion	Continuous measure of months	CPRD data
Physician diagnosed history of migraines or headaches	Binary variable indicating previous record (before TBI event) of headaches or migraines using codes provided in Appendix B	CPRD data
Learning disabilities	Binary variables indicating learning disabilities, as defined using codes in Appendix B	CPRD data
Attention deficit (hyperactivity) disorder	Binary variables indicating attention deficit (hyperactivity) disorder (before TBI event), as defined using codes in Appendix B	CPRD data

	As this is generally not diagnosed in very young children, this predictor variable will only be included in age stratified models for ages 5-10, 11-16 and 17-18	
Anxiety	Binary variables indicating previous history of anxiety (before TBI event), as defined using codes in Appendix B<br><br>As this is generally not diagnosed in young children, this predictor variable will only be included in age stratified models for ages 11-16 and 17-18	CPRD data
Depression	Binary variables indicating history of depression (before TBI event), as defined using codes in Appendix B<br><br>As this is generally not diagnosed in young children, this predictor variable will only be included in age stratified models for ages 11-16 and 17-18	CPRD data
Sleep disorders	Binary variables indicating history of sleep disorders (before TBI event), as defined using codes in Appendix B<br><br>As this is generally not diagnosed in very young children, this predictor variable will only be included in age stratified models for ages 5-10, 11-16 and 17-18	CPRD data

6. DATA SOURCES

Investigation of PCS prevalence, model development and internal validation will be conducted using routinely collected primary care data from Clinical Practice Research Datalink (CPRD) GOLD and Aurum. CPRD GOLD anonymised electronic primary care records for approximately 20 million patients registered at 968 participating general practices, using Vision® GP software, in the UK (June 2021 release notes). CPRD Aurum contains anonymised electronic primary care records for approximately 40 million patients registered at 1,491 participating general practices, using EMIS® GP software, in the UK (June 2021 release notes).

Secondary care data and socioeconomic status will be obtained via linkage to Hospital Episode Statistics Admitted Patient Care data (HES APC), HES Accident and Emergency (HES AE), HES Diagnostic Imaging Dataset (HES DID), practice level index of multiple deprivation and the patient-level indices of multiple deprivation (IMD) for patients.

7. STATISTICAL ANALYSES

7.1 Descriptive data

Continuous variables will be summarised using means and standard deviations (SD) (or medians and interquartile ranges (IQR) if the distribution is highly skewed), and categorical data will be summarised as numbers and percentages. Descriptive data will be stratified by the outcome (no PCS compared with PCS) and Chi-square tests will be used to test for differences between categorical variables and t-tests for continuous variables. Descriptive statistics and bivariate p values will be reported in **Table 1 (Appendix A)**.

7.2 Model development

Potential predictors will be identified using logistic regression models. Univariable analysis will assess associations between all predictor variables and the outcome variable. Continuous variables will be checked for non-linearity (using scatter plots) and if model assumptions are violated we will explore transforming the scale or identify the relationship (eg, quadratic) and include the relevant variable (eg, its square) in the model. We will also check for interactions between various predictor variables. If any predictor variables are strongly correlated factors (r>0.5) in exploratory analysis, one of these will be removed from the model. Any perfect predictors from any of the categorical variables will be combined with other categories.

We will fit the 'full model' with PCS as the outcome and including all candidate predictors listed in table 1 as covariates. Variable selection will be used using backwards elimination at p=0.1. Candidate prognostic factors dropped from the model will be discussed with experts and may be retained if considered important/clinically necessary (particularly if also identified in the scoping review).

We will assess a 'full' logistic regression model (containing all candidate predictors), Least Absolute Shrinkage and Selection Operator (LASSO), elastic net and ridge regression models and finally re-run the 'full' model using backwards elimination for variable selection at p=0.1. Estimates from the models are presented in **Table 2 (Appendix 1)**.

7.3 Internal validation of the model

Predictive performance of both the 'full' model and the most parsimonious model will be evaluated using bootstrapping. Bootstrapping methods will be used to test the internal validity of the model and identify model over-fitting. Models produced using bootstrapping will be compared with the original final model (difference between the two means of the two sets of C-statistics) to indicated optimism. Overall predictive accuracy will be assessed using R^2 and the Brier score which takes a value between 0 and 1, with lower scores indicating more accurate prediction. Discrimination is the ability of the model to distinguish between those with and without PCS. To assess discrimination we will report the C-statistic, with higher scores indicating better discrimination. Model indices are presented in **Table 3 (Appendix 1)**.

Calibration is the agreement between predicted and observed outcomes, also known as the calibration slope. Calibration will be explored by plotting the observed frequencies against the predicted probabilities in a graph, in 10 risk groups of equal

size, where an intercept of 0 and a slope of 1 indicates perfect calibration (**Figure 1, Appendix 1**).

7.3.1 Data presentation and analysis models

The outcome (binary indicator of PCS) will be summarised for the whole sample and by age group as frequencies and percentages. Outcomes will be compared using logistic regression models, and treatment comparisons presented as an odds ratios (OR), 95% CI and p-value.

Given the sensitive nature of this analysis, we will ensure that we follow good reporting practices, suppressing all non-zero counts below ten and any rates or proportions derived from these, unless they are in the unknown category. To minimise the need for suppression resulting in loss of data, we will aggregate data when possible.

7.3.2 Subgroup analyses

We will repeat analysis for different age groups, eg, school age groups, 1-4, 5-10, 11-15, 16-17, providing numbers of observations allows this number of categories (if not, small categories will be combined).

7.3.3 Sensitivity analyses

In addition to the main analysis, which will measure PCS as either a diagnosis of PCS or at least one PCS symptom within three months and one year after the TBI event, we will also run the following sensitivity analyses, using the final model only, changing the parameters of the PCS outcome measure:

- A PCS diagnosis or at least two PCS symptoms between three months and one year of the TBI event
- A PCS diagnosis or at least one PCS symptom between three months and three years of the TBI event
- A PCS diagnosis or at least two PCS symptoms between three months and three years of the TBI event

7.3.4 Missing data

For the outcome, and predictors (eg, ADHD, depression), if no record of it is found, then it will be assumed the patients did not have the condition.

Using CPRD data, it is unlikely that key patient descriptives such as age and gender will have missing data. As SEP is linked to GP practice, missing data are unlikely. It is anticipated that ethnicity will have missing data, however this is unlikely to be missing at random and will not be imputed. If missingness >10% it will be used only in sensitivity analysis.

7.4 Statistical software

All data management and analyses will be performed in Stata version 17 (9).

8. REFERENCES

1. National Institute for Health and Care Excellence. Head injury: assessment and early management. NICE Clinical Guidance 176. 2014.
2. Sariaslan A, Sharp D, D'Onofrio B, Larsson H, Fazel S. Long-Term Outcomes Associated with Traumatic Brain Injury in Childhood and Adolescence: A Nationwide Swedish Cohort Study of a Wide Range of Medical and Social Outcomes. PLOS Med. 2016;13(8):e1002103.
3. Laskowski R, Creed J, Raghupathi R. Pathophysiology of Mild TBI: Implications for Altered Signaling Pathways. In: Kobeissy F, editor. Brain Neurotrauma: Molecular, Neuropsychological, and Rehabilitation Aspects. Boca Raton (FL): CRC Press/Taylor & Francis; 2015.
4. Permenter C, Sherman A. Postconcussive Syndrome. In: StatPearls. Treasure Island (FL): StatPearls Publishing LLC; 2019.
5. Zemek R, Barrowman N, Freedman SB, Gravel J, Gagnon I, McGahern C, et al. Clinical risk score for persistent postconcussion symptomsamong children with acute concussion in the ED. JAMA - J Am Med Assoc [Internet]. 2016 Mar 8 [cited 2022 Aug 11];315(10):1014–25. Available from: https://jamanetwork.com/journals/jama/fullarticle/2499274
6. Babcock L, Byczkowski T, Wade SL, Ho M, Mookerjee S, Bazarian JJ. Predicting postconcussion syndrome after mild traumatic brain injury in children and adolescents who present to the emergency department. JAMA Pediatr [Internet]. 2013 Feb 1 [cited 2022 Aug 11];167(2):156–61. Available from: https://jamanetwork.com/journals/jamapediatrics/fullarticle/1486427
7. Boake C, McCauley S, Levin H. Diagnostic criteria for postconcussional syndrome after mild to moderate traumatic brain injury. J Neuropsychiatry Clin Neurosci [Internet]. 2005;17(3):350–6. Available from: https://neuro.psychiatryonline.org/doi/full/10.1176/jnp.17.3.350
8. Rubin DB, Schenker N. Multiple imputation in health-care databases: An overview and some applications. Stat Med [Internet]. 1991 Apr [cited 2016 Dec 2];10(4):585–98. Available from: http://doi.wiley.com/10.1002/sim.4780100410
9. StataCorp. Stata Statistical Software: Release 17. College Station, TX: StataCorp LLC; 2021.

APPENDIX A: SKELETON TABLES AND FIGURES

List tables and figures displayed in this SAP. These should all have been referenced elsewhere in the document. Examples of tables and figures which may be included are listed below.

**Tables**	
Table 1	Patient characteristics
Table 2	Full and variable selection predictive models
Table 3	Model performance
**Figures**	
Figure 1	Calibration plot

Table 1 Patient characteristics stratified by post-concussion syndrome

		No PCS (n=xx)		PCS (n=xx)		
		**n**	**%**	**n**	**%**	**p**
**Patient characteristics**						
Sex	Male					
	Female					
Age (years, months)						
Ethnicity	White					
	Black					
	Asian					
	Mixed					
	Other					
	missing					
IMD decile	Least deprived: 1					
	2					
	3					
	4					
	5					
	6					
	7					
	8					
	9					
	Most deprived: 10					
**Potential predictors**						
Prior concussion	No					
	Yes					
Time since last concussion (months)						
Physician diagnosed history of migraines or headaches	No					
	Yes					
Learning disabilities	No					
	Yes					
Attention deficit (hyperactive) disorder	No					
	Yes					
Anxiety	No					
	Yes					
Depression	No					
	Yes					
Sleep disorders	No					
	Yes					

Table 2: Predictive models

**Predictor variables**	**Full model (no variable selection) OR (95% CI)**	**LASSO model OR**	**Elastic net model OR**	**Ridge regression model OR**	**Full model (with backwards elimination) OR (95% CI)**

Table 3. Model performance

	**Full model (no variable selection)** OR (95% CI)	**LASSO model** OR	**Elastic net model** OR	**Ridge regression model** OR	**Full model (with backwards elimination)** OR (95% CI)
Brier scores					
Calibration intercept					
Calibration slope					
AUROC					

Figure 1: Calibration plot - observed risk of PCS and predicted risk of PCS

APPENDIX B: CRPD CODES

PCS Codes

PCS Codes, CPRD Gold			
**Med Code Id**	**Read code**	**Read term**	**Database build**
6139	F29y500	Chronic post-traumatic headache	Feb-09
6196	SC20.11	Late effect of head injury	Feb-09
10239	E2A2.00	Post-concussion syndrome	Feb-09
20582	Eu06212	[X]Post-traumatic brain syndrome	Feb-09
23710	Eu06.00	[X]Personality and behav disorder brain dis dam and dysfunct	Feb-09
24579	Eu06200	[X]Postconcussional syndrome	Feb-09
42704	E2A2.12	Post-head injury syndrome	Feb-09
49912	E2A2.11	Post-traumatic brain syndrome	Feb-09
99512	S604.00	Concussion with >24 hrs loss of consc without full recovery	May-10
103914	SC20100	Sequelae of injury to brain	Mar-12

PCS Codes, CPRD Aurum			
**Med Code Id**	**Term**	**Snomed CT Concept Id**	**Snomed CT Description Id**
2687391000006110.00	Postconcussion state	11764003.00	20313013.00
8826610000006117.00	Post concussional syndrome	40425004.00	882661000006117.00
3150771000006110.00	Postconcussion syndrome	40425004.00	67405010.00
492615014.00	Post-concussion syndrome	40425004.00	492615014.00
4230910000006111.00	Post-concussional syndrome	40425004.00	492616010.00
2169910000006113.00	Post-head injury syndrome	40425004.00	492616010.00

345367011.00	Chronic post-traumatic headache	230477005.00	345367011.00
502437100000006110.00	Post-concussional personality disorder	231530005.00	347015017.00
710666100000006110.00	Late effect of brain injury	429656004.00	2695751017.00
752582100000006110.00	Chronic post-concussion headache	699694000.00	2985894018.00
752583100000006110.00	Chronic post-concussive headache	699694000.00	2985442011.00
85354100000061 17.00	Post head injury syndrome	853541000006101.00	853541000006117.00
3636480016.00	Persistent headache due to and following injury of head	762354003.00	3636480016.00

PCS Codes, ICD10	
**ICD10 Code**	**Description**
F07	Personality and behavioural disorders due to brain disease, damage and dysfunction
F07.0	Organic personality disorder
F07.1	Postencephalitic syndrome
F07.2	Postconcussional syndrome
F07.8	Other organic personality and behavioural disorders due to brain disease, damage and dysfunction
F07.9	Unspecified organic personality and behavioural disorder due to brain disease, damage and dysfunction
G44.3	Chronic post-traumatic headache
G91.3	Post-traumatic hydrocephalus, unspecified

TBI Codes

TBI codes, CPRD GOLD			
**medcode**	**readcode**	**readterm**	**Database build**
27632	S643.00	Diffuse brain injury	Feb-09
10201	S64..11	Brain injury NOS	Feb-09
32214	S644.00	Focal brain injury	Feb-09
16553	S64..12	Head injury NOS	Feb-09
403	S64..13	Head injury	Feb-09
108055	SN55600	Non-accidental traumatic head injury to child	May-14

3008	S646.00	Head injury	Feb-09
102084	S010400	Closed #skull bse no intracranial injury, >24hr LOC+recovery	Apr-11
62977	S010.00	Closed fracture base of skull without intracranial injury	Feb-09
69737	S012.00	Open fracture base skull without mention intracranial injury	Feb-09
62743	S003z00	Open #skull vlt with intracranial injury + concussion unspec	Feb-09
111559	S011z00	Closed #skull bse + intracranial injury + concussion unspec	Dec-16
102718	S013z00	Open #skull bse + intracranial injury + concussion unspec	Aug-11
44273	Syu0E00	[X]Intracranial injury, unspecified	Feb-09
112563	S011200	Closed #skull bse + intracranial injury, <1hr loss of consc	Apr-18
64167	S003000	Open #skull vlt + intracranial injury, unspec state of consc	Feb-09
51308	S641.00	Intracranial injury NOS + open intracranial wound	Feb-09
33368	S64z.00	Intracranial injury NOS	Feb-09
94450	S010200	Closed #skull bse no intracranial injury, <1hr loss of consc	Feb-09
100796	S012200	Open #skull bse no intracranial injury, <1hr loss of consc	Nov-10
92354	S040.00	Mult #skull/face+other bones, closed, no intracranial injury	Feb-09
66312	S002100	Open #skull vlt no intracranial injury, no loss of consc	Feb-09
35456	S6...00	Intracranial injury excluding those with skull fracture	Feb-09
71725	S011400	Closed #skull bse + intracranial injury, >24hr LOC+recovery	Feb-09
98520	Syu0D00	[X]Other intracranial injuries	Feb-10
61357	S003600	Open #skull vlt + intracranial injury, LOC unspec duration	Feb-09
62841	S001600	Closed #skull vlt + intracranial injury, LOC unspec duration	Feb-09
72412	S001400	Closed #skull vlt + intracranial injury, >24hr LOC+recovery	Feb-09
73451	S013400	Open #skull bse + intracranial injury, >24hr LOC + recovery	Feb-09
73441	S003100	Open #skull vlt + intracranial injury, no loss of consc	Feb-09
65647	S645000	Intracranial injury with prolonged coma without open wound	Feb-09
97064	S013.00	Open fracture base of skull with intracranial injury	Jul-09
66765	S000300	Closed #skull vlt no intracranial injury, 1-24hr loss consc	Feb-09
113103	S000400	Closed #skull vlt no intracranial injury, >24hr LOC+recovery	Dec-18

35867	S630.12	Intracranial haematoma following injury	Feb-09
73790	S645100	Intracranial injury with prolonged coma with open wound	Feb-09
73206	S041.00	Mult #skull/face+other bones, closed + intracranial injury	Feb-09
58688	S010000	Closed #skull bse no intracranial injury, unspec state consc	Feb-09
50524	S000200	Closed #skull vlt no intracranial injury, <1hr loss of consc	Feb-09
66114	S002000	Open #skull vlt no intracranial injury, unspec state consc	Feb-09
67603	S033.00	Open fracture of skull NOS with intracranial injury	Feb-09
112568	S011100	Closed #skull bse + intracranial injury, no loss of consc	Apr-18
58957	S010100	Closed #skull bse no intracranial injury, no loss of consc	Feb-09
99018	S001100	Closed #skull vlt + intracranial injury, no loss of consc	Apr-10
17958	S001000	Closed #skull vlt + intracranial injury, unspec state consc	Feb-09
104726	S003400	Open #skull vlt + intracranial injury, >24hr LOC + recovery	Aug-12
99505	S013200	Open #skull bse + intracranial injury, <1hr loss of consc	May-10
96406	S010z00	Closed #skull bse no intracranial injury + concussion unspec	Apr-09
32298	S6z..00	Intracranial injury, excluding those with skull fracture NOS	Feb-09
27492	S031.00	Closed fracture of skull NOS with intracranial injury	Feb-09
62955	S000100	Closed #skull vlt no intracranial injury, no loss of consc	Feb-09
113378	S013000	Open #skull bse + intracranial injury, unspec state of consc	Feb-19
40393	S645.00	Intracranial injury with prolonged coma	Feb-09
45956	S011600	Closed #skull bse + intracranial injury, LOC unspec duration	Feb-09
98776	S001200	Closed #skull vlt + intracranial injury, <1hr loss of consc	Mar-10
59959	S001z00	Closed #skull vlt with intracranial injury+concussion unspec	Feb-09
57246	S002.00	Open fracture vault of skull without intracranial injury	Feb-09
47842	S000.00	Closed fracture vault of skull without intracranial injury	Feb-09
2864	S640.00	Intracranial injury NOS no open intracranial wound	Feb-09
94411	S032.00	Open #skull NOS without mention of intracranial injury	Feb-09
57644	S030.00	Closed fracture of skull NOS without intracranial injury	Feb-09
63679	S011.00	Closed fracture base of skull with intracranial injury	Feb-09
65724	S000000	Closed #skull vlt no intracranial injury, unspec state consc	Feb-09
73430	S000z00	Closed #skull vlt no intracranial injury + concussion unspec	Feb-09
93851	S001300	Closed #skull vlt + intracranial injury, 1-24hr loss consc	Feb-09

107041	S043.00	Mult #skull/face + other bones, open + intracranial injury	Oct-13
73411	S042.00	Mult #skull/face + other bones, open, no intracranial injury	Feb-09
105137	S011300	Closed #skull bse + intracranial injury, 1-24hr loss consc	Nov-12
51299	S003.00	Open fracture vault of skull with intracranial injury	Feb-09
27657	S001.00	Closed fracture vault of skull with intracranial injury	Feb-09
99282	S013600	Open #skull bse + intracranial injury, LOC unspec duration	May-10
34870	S64..00	Intracranial injury NOS	Feb-09
61388	S03..00	Other and unqualified skull fractures	Feb-09
23780	S03z.11	Depressed skull fracture NOS	Feb-09
39859	S04..12	Multiple skull fractures	Feb-09
57328	S03z.00	Skull fracture NOS	Feb-09
93458	S622z00	Subdural h'ge inj no open intracran wound+concussion unspec	Feb-09
106486	S620z00	Subarach h'ge inj no open intracran wnd + concussion unspec	Aug-13
106511	S621z00	Subarachnoid h'ge inj + open intracran wnd+concussion unspec	Aug-13
112954	S040z00	Closed #skull/face,mult,no intracran inj, concussion unspec	Oct-18
71866	S041z00	Closed #skull/face,mult + intracran inj, concussion unspec	Feb-09
105699	S61yz00	Brain cont + open intracranial wound + concussion unspec	Jan-13
66754	S640z00	Intracranial inj NOS no open intracran wnd+concussion unspec	Feb-09
89688	S030z00	Closed #skull NOS no intracranial inj + concussion unspec	Feb-09
113980	S031z00	Closed #skull NOS + intracranial inj + concussion unspec	Jul-19
65532	S032z00	Open #skull NOS no intracranial inj + concussion unspec	Feb-09
94409	S610z00	Cortex cont no open intracranial wnd + concussion unspec	Feb-09
96731	S624z00	Extradural h'ge inj no open intracran wnd+concussion unspec	Apr-09
56509	S641z00	Intracranial inj NOS + open intracran wnd+concussion unspec	Feb-09
104820	S61xz00	Brain cont no open intracranial wound + concussion unspec	Aug-12
69209	S611.00	Open cerebral contusion	Feb-09
12770	S61..00	Cerebral laceration and contusion	Feb-09
42692	S61z.00	Cerebral laceration and contusion NOS	Feb-09

33455	S610.00	Closed cerebral contusion	Feb-09
48325	S61..11	Cerebellar laceration and contusion	Feb-09
7862	S629.00	Traumatic subdural haematoma	Feb-09
96677	S629100	Traumatic subdural haematoma with open intracranial wound	Apr-09
53980	S629000	Traumatic subdural haematoma without open intracranial wound	Feb-09
97911	S62A000	Traumatic extradural haemat without open intracranial wound	Oct-09
109369	S62A100	Traumatic extradural haematoma with open intracranial wound	Mar-15
18411	S62A.00	Traumatic extradural haematoma	Feb-09
96732	S624100	Extradural h'ge inj no open intracranial wnd + no loss consc	Apr-09
73541	S624000	Extradural h'ge inj no open intracranial wnd + unspec consc	Feb-09
27661	S62..11	Extradural haemorrhage following injury	Feb-09
114667	S624500	Extradural h'ge inj no open intracran wnd+>24hr LOC-restored	Feb-20
113416	S624300	Extradural h'ge inj no open intracran wnd+1-24hr loss consc	Mar-19
114084	S625000	Extradural h'ge inj + open intracranial wnd + unspec consc	Aug-19
114413	S611000	Cortex cont + open intracranial wound + unspec state consc	Dec-19
99072	S642100	Traumatic cerebral oedema with open intracranial wound	Apr-10
113300	S621100	Subarachnoid h'ge inj + open intracranial wound + no LOC	Feb-19
94076	S613.00	Cortex laceration with open intracranial wound	Feb-09
71963	S642000	Traumatic cerebral oedema without open intracranial wound	Feb-09
61318	S613400	Cortex lacn + open intracranial wound + >24hr LOC + recovery	Feb-09
68560	S61x000	Brain cont no open intracranial wound + unspec state consc	Feb-09
113577	S622200	Subdural h'ge inj no open intracranial wound+<1hr loss consc	Apr-19
114601	S61x100	Brain cont no open intracranial wound + no loss of consc	Dec-19
56638	S630.00	Other cerebral h'ge after injury no open intracranial wound	Feb-09
108089	S612000	Cortex lacn no open intracranial wound + unspec state consc	May-14
112879	S61x200	Brain cont no open intracranial wound + <1hr loss of consc	Sep-18

52391	S641100	Intracranial inj NOS + open intracranial wound+no loss consc	Feb-09
100875	S61x600	Brain cont no open intracranial wound + LOC unspec duration	Nov-10
114652	S622100	Subdural h'ge inj no open intracranial wound+no loss consc	Feb-20
107337	S647.00	Acquired brain injury	Dec-13
6303	S640111	Minor head injury	Feb-09
8314	S646000	Minor head injury	Feb-09
28416	S60z.00	Concussion NOS	Feb-09
28079	S600.00	Concussion with no loss of consciousness	Feb-09
51682	S602.00	Concussion with 1-24 hours loss of consciousness	Feb-09
99512	S604.00	Concussion with >24 hrs loss of consc without full recovery	May-10
15558	S601.00	Concussion with less than 1 hour loss of consciousness	Feb-09
38445	S605.00	Concussion with loss of consciousness of unspec duration	Feb-09
61239	S603.00	Concussion with >24 hrs loss of consc with full recovery	Feb-09
1520	S60..00	Concussion	Feb-09
7017	7004300	Evacuation of intracerebral haematoma NEC	Feb-09
4917	7017000	Evacuation of subdural haematoma	Feb-09
4107	7032000	Evacuation of extradural haematoma	Feb-09

TBI codes, CPRD Aurum

Med Code Id	Term	Snomed CT Concept Id	Snomed CT Description Id
4396301000006119	Traumatic brain injury with moderate loss of consciousness	127300000	588013
7380591000006114	Traumatic brain injury with loss of consciousness one hour or more	450569000	2915718018
4396321000006112	Traumatic brain injury with no loss of consciousness	127302008	590014
4174151000006111	MTBI - Mild traumatic brain injury	110030002	3034714010
4396281000006118	Traumatic brain injury with loss of consciousness	127298000	586012
4396291000006115	Traumatic brain injury with brief loss of consciousness	127299008	587015
3058171000006119	Contusional brain injury	34663006	1228197014
4174161000006113	Mild traumatic brain injury	110030002	3034729012
1808531000006113	Traumatic brain injury	127295002	1808531000006113
3783541000006116	Lacerating brain injury	78914008	503949015
409932013	Minor head injury	274164006	409932013
1874181000006117	Child young persons injury - head injury (other)	1874181000006101	1874181000006117
2289801000000113	Non-accidental traumatic head injury to child	700506009	2990833010
6048871000006116	Moderate head injury	314661000	459050013
698741000006119	Minor head injury	274164006	409932013
1874171000006115	Child young persons injury - head injury (concussion)	1874171000006104	1874171000006115
459051012	Major head injury	314662007	459051012
136462012	Head injury	82271004	136462012
1954631000006117	Head injury (secondary group)	1954631000006101	1954631000006117
390461014	Head injury NOS	82271004	136462012
1984181000006119	Manchester triage - Head injury	1984181000006103	1984181000006119
817721000006116	Head injury	82271004	136462012

3838151000006112	HI - Head injury	82271004	504994017
3381941000006112	Head injury, without skull fracture	54355006	90342012
907031000006111	[RFC] Head injury	907031000006107	907031000006111
318117015	Closed fracture vault of skull with intracranial injury	207687004	318117015
318135012	Open fracture vault of skull with intracranial injury	207705002	318135012
52931000006111	Open #skull vlt + intracranial injury, >24hr LOC + recovery	207710003	318140016
318285013	Open fracture of skull NOS with intracranial injury	371161001	1209863015
325304010	[X]Other intracranial injuries	127296001	584010
263841000006116	Open #skull vlt no intracranial injury, 1-24hr loss of consc	207696004	318126017
263781000006117	Open #skull vlt + intracranial injury, no loss of consc	207707005	318137016
264211000006117	Open #skull bse no intracranial injury, <1hr loss of consc	24063002	40395011
263881000006110	Open #skull vlt with intracranial injury + concussion unspec	207705002	318135012
264131000006117	Open #skull bse + intracranial injury, >24hr LOC + recovery	207748008	318192013
264141000006110	Open #skull bse + intracranial injury, 1-24hr loss of consc	207747003	318191018
263771000006115	Open #skull vlt + intracranial injury, LOC unspec duration	207705002	318135012
263811000006115	Open #skull vlt no intracranial injury + concussion unspec	207696004	318126017
263821000006111	Open #skull vlt no intracranial injury, <1hr loss of consc	207696004	318126017
259601000006113	Open fracture base of skull with intracranial injury	111607004	178855010
263791000006119	Open #skull vlt + intracranial injury, unspec state of consc	207705002	318135012
264201000006115	Open #skull bse no intracranial injury + concussion unspec	24063002	40395011
264241000006118	Open #skull bse no intracranial injury, LOC unspec duration	24063002	40395011
264451000006115	Open #skull bse + intracranial injury + concussion unspec	111607004	178855010
52941000006118	Open #skull vlt + intracranial injury, 1-24hr loss of consc	207709008	318139018
263871000006112	Open #skull vlt no intracranial injury, unspec state consc	207696004	318126017

26412100 0006115	Open #skull bse + intracranial injury, <1hr loss of consc	207746007	31819001 7
26415100 0006112	Open #skull bse + intracranial injury, LOC unspec duration	111607004	17885501 0
26443100 0006110	Open #skull vlt + intracranial injury, <1hr loss of consc	207708000	31813801 4
55998100 0006117	Closed #skull bse + intracranial injury, <1hr loss of consc	207728001	31817201 8
56006100 0006113	Closed #skull bse no intracranial injury, <1hr loss of consc	27644009	46274013
56042100 0006110	Closed #skull vlt with intracranial injury+concussion unspec	207687004	31811701 5
56001100 0006110	Closed #skull bse + intracranial injury, LOC unspec duration	111603000	17885301 5
56005100 0006111	Closed #skull bse no intracranial injury + concussion unspec	27644009	46274013
56037100 0006115	Closed #skull vlt no intracranial injury, <1hr loss of consc	69866009	11602901 6
55999100 0006119	Closed #skull bse + intracranial injury, >24hr LOC+recovery	207730004	31817401 7
56008100 0006115	Closed #skull bse no intracranial injury, 1-24hr loss consc	27644009	46274013
55997100 0006115	Closed #skull bse + intracranial injury + concussion unspec	111603000	17885301 5
56036100 0006110	Closed #skull vlt no intracranial injury + concussion unspec	69866009	11602901 6
56038100 0006117	Closed #skull vlt no intracranial injury, >24hr LOC+recovery	69866009	11602901 6
56165100 0006113	Closed fracture base of skull with intracranial injury	111603000	17885301 5
56031100 0006112	Closed #skull vlt + intracranial injury, LOC unspec duration	207687004	31811701 5
56032100 0006116	Closed #skull vlt + intracranial injury, no loss of consc	207689001	31811901 7
56041100 0006119	Closed #skull vlt no intracranial injury, unspec state consc	69866009	11602901 6
69614100 0006119	Mult #skull/face+other bones, closed + intracranial injury	5468008	10127011
69615100 0006117	Mult #skull/face+other bones, closed, no intracranial injury	5468008	10127011
56000100 0006112	Closed #skull bse + intracranial injury, 1-24hr loss consc	207729009	31817301 1
56030100 0006114	Closed #skull vlt + intracranial injury, 1-24hr loss consc	207691009	31812101 0
56519100 0006110	Closed fracture vault of skull without intracranial injury	69866009	11602901 6
69613100 0006112	Mult #skull/face + other bones, open, no intracranial injury	5468008	10127011

318266010	Closed fracture of skull NOS with intracranial injury	111613008	178858012
4198041000006111	Closed skull fracture with intracranial injury	111613008	4198041000006111
124840510000006114	Closed fracture of skull NOS with intracranial injury	371162008	563781000006112
264221000006113	Open #skull bse no intracranial injury, >24hr LOC+recovery	24063002	40395011
560021000006119	Closed #skull bse + intracranial injury, no loss of consc	207727006	318171013
264231000006111	Open #skull bse no intracranial injury, 1-24hr loss of consc	24063002	40395011
560281000006110	Closed #skull vlt + intracranial injury, <1hr loss of consc	207690005	318120011
561661000006110	Closed fracture base of skull without intracranial injury	27644009	46274013
560091000006117	Closed #skull bse no intracranial injury, no loss of consc	27644009	46274013
563791000006110	Closed fracture of skull NOS without intracranial injury	71642004	119023019
259611000006111	Open fracture base skull without mention intracranial injury	24063002	40395011
263861000006117	Open #skull vlt no intracranial injury, no loss of consc	207696004	318126017
560391000006119	Closed #skull vlt no intracranial injury, 1-24hr loss consc	69866009	116029016
560401000006117	Closed #skull vlt no intracranial injury, no loss of consc	69866009	116029016
264161000006114	Open #skull bse + intracranial injury, no loss of consc	207745006	318189014
696121000006114	Mult #skull/face + other bones, open + intracranial injury	5468008	10127011
264411000006116	Open #skull NOS without mention of intracranial injury	371161001	1209863015
560071000006118	Closed #skull bse no intracranial injury, >24hr LOC+recovery	27644009	46274013
264171000006119	Open #skull bse + intracranial injury, unspec state of consc	111607004	178855010
560331000006118	Closed #skull vlt + intracranial injury, unspec state consc	207687004	318117015
263851000006119	Open #skull vlt no intracranial injury, LOC unspec duration	207696004	318126017
263831000006114	Open #skull vlt no intracranial injury, >24hr LOC+recovery	207696004	318126017
264251000006116	Open #skull bse no intracranial injury, no loss of consc	24063002	40395011

318126017	Open fracture vault of skull without intracranial injury	207696004	318126017
560101000006111	Closed #skull bse no intracranial injury, unspec state consc	24063002	40395011
560291000006113	Closed #skull vlt + intracranial injury, >24hr LOC+recovery	207692002	318122015
320931016	Intracranial injury with prolonged coma	210039000	320931016
455459010	Intracranial injury with prolonged coma without open wound	311827003	455459010
486896012	Intracranial haematoma following injury	37955001	486896012
402934010	Intracranial injury NOS	127296001	584010
325305011	[X]Intracranial injury, unspecified	127296001	584010
768881000006110	Intracranial injury excluding those with skull fracture	54355006	90339018
768961000006113	Intracranial injury, excluding those with skull fracture NOS	54355006	90339018
768891000006113	Intracranial injury NOS	127296001	584010
7378281000006118	Intracranial hemorrhage following injury	450410005	2915467017
12727601000006112	Intracranial injury NOS	684491000000102	1499081000000113
12757871000006118	Intracranial hemorrhage following injury	82894007	137497019
12716261000006116	Intracranial haemorrhage following injury	82894007	505321018
768911000006110	Intracranial injury NOS + open intracranial wound	127296001	584010
7378271000006116	Intracranial haemorrhage following injury	450410005	2915426010
768921000006119	Intracranial injury NOS no open intracranial wound	127296001	584010
455461018	Intracranial injury with prolonged coma with open wound	311829000	455461018
3111241000006113	Intracranial hematoma following injury	37955001	63313013
318256019	Other and unqualified skull fractures	71642004	119023019
613741000006112	Depressed skull fracture NOS	71642004	119023019

69498100 0006118	Multiple skull fractures	49128003	81844012
40282901 2	Skull fracture NOS	71642004	11902301 9
12723091 00000611 8	Skull fracture NOS	544741000 000101	12145210 00000115
26433100 0006115	Open #skull NOS no intracranial inj + concussion unspec	371161001	12098630 15
26401100 0006112	Open #skull/face,mult,no intracran inj, concussion unspec	5468008	10127011
52440100 0006115	Brain cont no open intracranial wound + concussion unspec	269144002	40292101 4
52432100 0006119	Brain cont + open intracranial wound + concussion unspec	269144002	40292101 4
82336100 0006114	Hind brain cont + open intracranial wnd + concussion unspec	209881009	32065201 0
76886100 0006117	Intracranial inj NOS no open intracran wnd+concussion unspec	127296001	584010
76932100 0006113	Intracranial inj NOS + open intracran wnd+concussion unspec	127296001	584010
65998100 0006114	Extradural h'ge inj + open intracran wnd+concussion unspec	65189006	10833201 2
39711000 006113	Oth cereb h'ge inj + open intracran wnd+concussion unspec	450418003	29152880 12
12331100 0006115	Subarach h'ge inj no open intracran wnd + concussion unspec	28048009	46957015
12408100 0006114	Subdural h'ge inj + open intracranial wnd+concussion unspec	209956005	32077001 5
39751000 006114	Oth cereb h'ge inj no open intracran wnd+concussion unspec	450418003	29152880 12
12335100 0006119	Subarachnoid h'ge inj + open intracran wnd+concussion unspec	5251007	9804011
82363100 0006110	Hind brain lacn no open intracranial wnd + concussion unspec	78914008	13094201 5
59777100 0006117	Cortex cont no open intracranial wnd + concussion unspec	209834008	32060201 8
56054100 0006119	Closed #skull/face,mult + intracran inj, concussion unspec	5468008	10127011
12415100 0006119	Subdural h'ge inj no open intracran wound+concussion unspec	209947002	32075201 8
56019100 0006116	Closed #skull NOS no intracranial inj + concussion unspec	371162008	12098640 14
26389100 0006113	Open #skull/face, mult + intracran inj + concussion, unspec	5468008	10127011
82355100 0006116	Hind brain lacn + open intracranial wnd + concussion unspec	209900006	32067201 7

660071000006113	Extradural h'ge inj no open intracran wnd+concussion unspec	262949005	391026017
597981000006117	Cortex lacn no open intracranial wound + concussion unspec	78914008	130942015
823431000006112	Hind brain cont no open intracranial wnd + concussion unspec	209871005	320639017
264261000006119	Open #skull NOS + intracranial inj + concussion unspec	371161001	1209863015
560561000006115	Closed #skull/face,mult,no intracran inj, concussion unspec	5468008	10127011
597901000006114	Cortex lacn + open intracranial wound + concussion unspec	59748008	99251014
560111000006114	Closed #skull NOS + intracranial inj + concussion unspec	371162008	1209864014
597711000006114	Cortex cont + open intracranial wound + concussion unspec	209843004	320611018
103238011	Concussion with no loss of consciousness	62106007	103238011
320593012	Concussion with 1-24 hours loss of consciousness	209828001	320593012
578281000006118	Concussion with >24 hrs loss of consc with full recovery	209829009	320594018
578291000006115	Concussion with >24 hrs loss of consc without full recovery	209830004	320595017
578321000006112	Concussion with loss of consciousness of unspec duration	62564004	103980016
3517181000006112	Concussion with loss of consciousness	62564004	103979019
4174111000006110	Concussion injury of brain	110030002	345267014
320597013	Concussion NOS	110030002	174751018
320592019	Concussion with less than 1 hour loss of consciousness	209827006	320592019
202903013	Concussion	110030002	202903013
320611018	Open cerebral contusion	209843004	320611018
39851000006115	Oth cerebral laceration/contusion no open intracranial wound	269144002	402921014
39841000006117	Oth cerebral laceration/contusion + open intracranial wound	269144002	402921014
320602018	Closed cerebral contusion	209834008	320602018
402921014	Cerebral laceration and contusion	269144002	402921014
390673018	Cerebellar laceration and contusion	262687004	390673018

896521000006112	Cerebral laceration/contusion	269144002	896521000006112
12731781000006118	Cerebral laceration and contusion NOS	566951000000103	1260521000000110
320699015	Cerebral laceration and contusion NOS	269144002	402921014
459514014	Traumatic subdural haematoma with open intracranial wound	315047001	459514014
459511018	Traumatic subdural haematoma without open intracranial wound	315046005	459511018
5459221000006115	Traumatic cranial subdural haematoma	262952002	391036013
5459261000006114	Traumatic subdural haematoma	262952002	2913152010
5459231000006117	Traumatic intracranial subdural haematoma	262952002	391037016
391035012	Traumatic subdural haematoma	262952002	2912898019
860210000006119	Traumatic extradural haemat without open intracranial wound	315048006	459515010
391028016	Traumatic extradural haematoma	262949005	391028016
459518012	Traumatic extradural haematoma with open intracranial wound	315049003	459518012
5459201000006113	Traumatic intracranial extradural haematoma	262949005	2916480013
320672017	Hind brain laceration with open intracranial wound	209900006	320672017
320621014	Cortex laceration without mention of open intracranial wound	78914008	130942015
124191000006113	Subdural h'ge inj no open intracranial wound+no loss consc	209947002	320752018
124061000006116	Subdural h'ge inj + open intracran wound+>24hr LOC +recovery	52902005	496480019
124101000006118	Subdural h'ge inj + open intracranial wound+<1hr loss consc	29635000	484345019
124111000006115	Subdural h'ge inj + open intracranial wound+no loss consc	209958006	320775013
524391000006117	Brain cont no open intracranial wound + 1-24hr loss of consc	34663006	57855014
524351000006111	Brain cont + open intracranial wound + unspec state consc	269144002	402921014
524301000006112	Brain cont + open intracranial wound + >24hr LOC + recovery	209923009	320695014
524421000006113	Brain cont no open intracranial wound + no loss of consc	34663006	57855014

524331000006116	Brain cont + open intracranial wound + LOC unspec duration	269144002	402921014
524381000006115	Brain cont no open intracranial wound + >24hr LOC + recovery	34663006	57855014
524411000006117	Brain cont no open intracranial wound + LOC unspec duration	269144002	402921014
597731000006115	Cortex cont + open intracranial wound + unspec state consc	87888006	145700010
597871000006114	Cortex lacn + open intracranial wound + <1hr loss of consc	209865004	320633016
597991000006119	Cortex lacn no open intracranial wound + no loss of consc	78914008	130942015
597881000006112	Cortex lacn + open intracranial wound + >24hr LOC + recovery	209867007	320635011
597891000006110	Cortex lacn + open intracranial wound + 1-24hr loss of consc	209866003	320634010
524431000006111	Brain cont no open intracranial wound + unspec state consc	34663006	57855014
823511000006117	Hind brain laceration without open intracranial wound	78914008	130942015
597921000006116	Cortex lacn + open intracranial wound + unspec state consc	59748008	99251014
598001000006112	Cortex lacn no open intracranial wound + unspec state consc	78914008	130942015
660021000006112	Extradural h'ge inj + open intracranial wound+no loss consc	209978003	320817017
597911000006112	Cortex lacn + open intracranial wound + no loss of consc	209864000	320632014
597691000006111	Cortex cont + open intracranial wound + <1hr loss of consc	209847003	320615010
769361000006119	Intracranial inj NOS + open intracranial wound+no loss consc	127296001	584010
4854181000006114	Subdural haemorrhage following injury without open intracranial wound	209947002	2915859018
3928351000006115	Cortex contusion with open intracranial wound	87888006	145700010
1190361100000061 7	Subarachnoid hemorrhage following injury with open intracranial wound AND moderate loss of consciousness (1-24 hours)	87253004	144684019
4854241000006112	Subdural haemorrhage following injury with open intracranial wound	209956005	2916362015
4854261000006111	Subdural hemorrhage following injury with open intracranial wound, with no loss of consciousness	209958006	320774012
3901101000006118	Extradural haemorrhage following injury with open intracranial wound AND prolonged loss of consciousness (more than 24 hours) without return to pre-existing conscious level	86182004	3901101000006118

33578810 00006110	Subdural hemorrhage following injury with open intracranial wound AND prolonged loss of consciousness (more than 24 hours) AND return to pre-existing conscious level	52902005	88030010
60533510 00006112	Traumatic extradural hematoma with open intracranial wound	315049003	45951701 9
39185910 00006111	Subarachnoid haemorrhage following injury with open intracranial wound AND moderate loss of consciousness (1-24 hours)	87253004	39185910 00006111
60532910 00006112	Traumatic subdural hematoma without open intracranial wound	315046005	45951201 3
60099110 00006116	Traumatic cerebral edema with open intracranial wound	311826007	45545701 2
48543010 00006119	Extradural hemorrhage following injury with open intracranial wound, with no loss of consciousness	209978003	32081801 0
34686910 00006111	Subarachnoid hemorrhage following injury with open intracranial wound AND brief loss of consciousness (less than one hour)	59633005	99064015
34689810 00006117	Extradural hemorrhage following injury with open intracranial wound AND moderate loss of consciousness (1-24 hours)	59648004	99099016
36251510 00006114	Subarachnoid haemorrhage following injury with open intracranial wound AND prolonged loss of consciousness (more than 24 hours) without return to pre-existing conscious level	69178005	36251510 00006114
48541210 00006110	Subarachnoid hemorrhage following injury with open intracranial wound, with no loss of consciousness	209940000	32073801 5
29502310 00006111	Subarachnoid haemorrhage following injury without open intracranial wound	28048009	29502310 00006111
11903641 00000611 8	Subdural hemorrhage following injury with open intracranial wound AND moderate loss of consciousness (1-24 hours)	90165008	14946201 1
12342100 0006117	Subarachnoid h'ge inj no open intracran wound + 1-24hr LOC	28048009	46957015
35791000 006117	Other cerebral h'ge after injury + open intracranial wound	450418003	29152880 12
12336100 0006117	Subarachnoid h'ge inj + open intracran wound + unspec consc	5251007	9804011
12409100 0006112	Subdural h'ge inj + open intracranial wound + unspec consc	209956005	32077001 5
11923791 00000611 4	Subarachnoid hemorrhage following injury without open intracranial wound	28048009	46957015
36941410 00006116	Subarachnoid haemorrhage following injury with open intracranial wound AND prolonged loss of consciousness (more than 24 hours) AND return to pre-existing conscious level	73439007	36941410 00006116

6053311000006111	Traumatic subdural hematoma with open intracranial wound	315047001	459513015
597721000006118	Cortex cont + open intracranial wound + no loss of consc	209845006	320613015
4854231000006119	Subdural hemorrhage following injury with open intracranial wound	209956005	2915474010
119246410000006113	Extradural hemorrhage following injury with open intracranial wound AND prolonged loss of consciousness (more than 24 hours) without return to pre-existing conscious level	86182004	142911018
3559941000006115	Extradural haemorrhage following injury with open intracranial wound	65189006	3559941000006115
119035910000006111	Subarachnoid hemorrhage following injury with open intracranial wound AND prolonged loss of consciousness (more than 24 hours) AND return to pre-existing conscious level	73439007	121954015
4198411000006116	Subdural hemorrhage following injury with open intracranial wound and prolonged loss of consciousness (more than 24 hours) and without return to pre-existing conscious level	111677000	61538012
3965681000006115	Subdural haemorrhage following injury with open intracranial wound AND moderate loss of consciousness (1-24 hours)	90165008	3965681000006115
3196371000006118	Extradural hemorrhage following injury without open intracranial wound	43216008	72101019
123431000006119	Subarachnoid h'ge inj no open intracran wound + unspec consc	28048009	46957015
123371000006112	Subarachnoid h'ge inj + open intracran wound+<1hr loss consc	59633005	498500014
4854171000006111	Subdural hemorrhage following injury without open intracranial wound	209947002	2915477015
524371000006118	Brain cont no open intracranial wound + <1hr loss of consc	34663006	57855014
119246610000006112	Extradural hemorrhage following injury with open intracranial wound AND brief loss of consciousness (less than one hour)	73308006	121738013
823401000006116	Hind brain cont + open intracranial wound + <1hr loss consc	209884001	320655012
2976661000006116	Subdural hemorrhage following injury with open intracranial wound AND brief loss of consciousness (less than one hour)	29635000	49572012
3692151000006113	Extradural haemorrhage following injury with open intracranial wound AND brief loss of consciousness (less than one hour)	73308006	3692151000006113
119036010000006115	Subarachnoid hemorrhage following injury with open intracranial wound AND prolonged loss of consciousness (more than 24 hours) without return to pre-existing conscious level	69178005	114903015

6009891000006118	Traumatic cerebral edema without open intracranial wound	311825006	455456015
35801000006116	Other cerebral h'ge after injury no open intracranial wound	450418003	2915288012
524291000006111	Brain cont + open intracranial wound + <1hr loss of consc	209921006	320693019
3825541000006114	Extradural haemorrhage following injury with open intracranial wound AND prolonged loss of consciousness (more than 24 hours) AND return to pre-existing conscious level	81520002	3825541000006114
3928361000006118	Contusion of cerebral cortex with open intracranial wound	87888006	145701014
2583451000006111	Subarachnoid hemorrhage following injury with open intracranial wound	5251007	9804011
6053331000006117	Traumatic extradural hematoma without open intracranial wound	315048006	459516011
124181000006110	Subdural h'ge inj no open intracranial wound+<1hr loss consc	209947002	320752018
123381000006110	Subarachnoid h'ge inj + open intracranial wound + no LOC	209940000	320739011
597701000006111	Cortex cont + open intracranial wound + 1-24hr loss of consc	209848008	320616011
524311000006110	Brain cont + open intracranial wound + 1-24hr loss of consc	209922004	320694013
524341000006114	Brain cont + open intracranial wound + no loss of consc	209920007	320692012
455455016	Traumatic cerebral oedema without open intracranial wound	311825006	455455016
1190268100000611 7	Extradural hemorrhage following injury with open intracranial wound	65189006	108332012
1192465100000611 0	Extradural hemorrhage following injury with open intracranial wound AND prolonged loss of consciousness (more than 24 hours) AND return to pre-existing conscious level	81520002	135232013
823411000006118	Hind brain cont + open intracranial wound + no loss consc	209883007	320654011
99251014	Cortex laceration with open intracranial wound	59748008	99251014
455458019	Traumatic cerebral oedema with open intracranial wound	311826007	455458019
597971000006115	Cortex lacn no open intracranial wound + <1hr loss of consc	78914008	130942015
2273491000000112	Acquired brain injury	702632000	3005402014
4396241000006112	Acquired brain injury	127294003	2273491000000112
390682012	Diffuse brain injury	262693007	390682012

54558610 00006111	Diffuse axonal brain injury	262693007	39068301 9
32093001 5	Focal brain injury	210038008	32093001 5
39067201 1	Brain injury NOS	127294003	47410301 6
43962110 00006113	Brain tissue injury	127294003	47410101 9
81764100 0006117	Head and neck superficial nerve injury	269255007	40305501 6
81774100 0006111	Head injury - nursing supervis	82271004	13645901 4
40305501 6	Head and neck superficial nerve injury	269255007	40305501 6
36480610 00006116	Elevation of skull fracture	70590006	11725901 3
36480510 00006118	Elevation of skull fracture fragments	70590006	11725801 7
26569201 1	Evacuation of intracerebral haematoma NEC	10458001	27942901 4
49851601 1	Evacuation of subdural haematoma	59712006	49852001 0
34698510 00006111	Evacuation of subdural haematoma	59712006	49851601 1
34698910 00006117	Subdural haematoma evacuation	59712006	49851901 6
64954100 0006113	Evacuation of extradural haematoma	171713001	26596801 7
46456410 00006117	Evacuation of intracranial extradural haematoma	171713001	26596801 7
38505310 00006110	EDH - Extradural haematoma	82999001	12169990 17

**TBI codes, ICD10**	
**ICD10_Code**	**DESCRIPTION**
S02	Fracture of skull and facial bones
S020	Fracture of vault of skull
S021	Fracture of base of skull
S022	Fracture of nasal bones
S023	Fracture of orbital floor
S024	Fracture of malar and maxillary bones
S025	Fracture of tooth
S026	Fracture of mandible
S027	Multiple fractures involving skull and facial bones

S028	Fractures of other skull and facial bones
S029	Fracture of skull and facial bones, part unspecified
S06	Intracranial injury
S060	Concussion
S061	Traumatic cerebral oedema
S062	Diffuse brain injury
S063	Focal brain injury
S064	Epidural haemorrhage
S065	Traumatic subdural haemorrhage
S066	Traumatic subarachnoid haemorrhage
S067	Intracranial injury with prolonged coma
S068	Other intracranial injuries
S069	Intracranial injury, unspecified
S07	Crushing injury of head
S070	Crushing injury of face
S071	Crushing injury of skull
S078	Crushing injury of other parts of head
S079	Crushing injury of head, part unspecified
S08	Traumatic amputation of part of head
S080	Avulsion of scalp
S081	Traumatic amputation of ear
S088	Traumatic amputation of other parts of head
S089	Traumatic amputation of unspecified part of head
S090	Injury of blood vessels of head, not elsewhere classified
S092	Traumatic rupture of ear drum
S097	Multiple injuries of head
T020	Fractures involving head with neck
T040	Crushing injuries involving head with neck
T060	Injuries of brain and cranial nerves with injuries of nerves and spinal cord at neck level
S00	Superficial injury of head
S000	Superficial injury of scalp
S001	Contusion of eyelid and periocular area
S002	Other superficial injuries of eyelid and periocular area
S003	Superficial injury of nose
S004	Superficial injury of ear
S005	Superficial injury of lip and oral cavity
S007	Multiple superficial injuries of head
S008	Superficial injury of other parts of head
S009	Superficial injury of head, part unspecified
S01	Open wound of head
S010	Open wound of scalp

S011	Open wound of eyelid and periocular area
S012	Open wound of nose
S013	Open wound of ear
S014	Open wound of cheek and temporomandibular area
S015	Open wound of lip and oral cavity
S017	Multiple open wounds of head
S018	Open wound of other parts of head
S019	Open wound of head, part unspecified
S09	Other and unspecified injuries of head
S091	Injury of muscle and tendon of head
S098	Other specified injuries of head
S099	Unspecified injury of head
T000	Superficial injuries involving head with neck
T010	Open wounds involving head with neck

**TBI codes, A&E diagnosis codes (diagnosis condition code + anatomical area code + anatomical side code)**				
**Diagnosis condition**		**Anatomical area**		**Anatomical side**
**code**	**description**	**code**	**description**	**code**
**04**	Head injury	**any**		**any**
**041**	Concussion	**any**		**any**
**042**	Other head injury	**any**		**any**
**05**	Dislocation/fracture/joint injury/amputation	**02**	Head	**any**
**05**	Dislocation/fracture/joint injury/amputation	**03**	Face	**any**
**05**	Dislocation/fracture/joint injury/amputation	**04**	Eye	**any**
**05**	Dislocation/fracture/joint injury/amputation	**05**	Ear	**any**
**05**	Dislocation/fracture/joint injury/amputation	**06**	Nose	**any**
**05**	Dislocation/fracture/joint injury/amputation	**07**	Mouth,Jaw,Teeth	**any**
**05**	Dislocation/fracture/joint injury/amputation	**36**	Multiple site	**any**
**052**	Open fracture	**02**	Head	**any**
**052**	Open fracture	**03**	Face	**any**
**052**	Open fracture	**04**	Eye	**any**
**052**	Open fracture	**05**	Ear	**any**
**052**	Open fracture	**06**	Nose	**any**
**052**	Open fracture	**07**	Mouth,Jaw,Teeth	**any**
**053**	Open fracture	**36**	Multiple site	**any**
**053**	Closed fracture	**02**	Head	**any**
**053**	Closed fracture	**03**	Face	**any**
**053**	Closed fracture	**04**	Eye	**any**
**053**	Closed fracture	**05**	Ear	**any**
**053**	Closed fracture	**06**	Nose	**any**
**053**	Closed fracture	**07**	Mouth,Jaw,Teeth	**any**

**054**	Closed fracture	**36**	Multiple site	**any**
**051**	Dislocation	**07**	Mouth,Jaw,Teeth	**any**
**052**	Dislocation	**36**	Multiple site	**any**
**021**	Contusion	**02**	Head	**any**
**022**	Contusion	**03**	Face	**any**
**023**	Contusion	**04**	Eye	**any**
**024**	Contusion	**05**	Ear	**any**
**025**	Contusion	**06**	Nose	**any**
**026**	Contusion	**07**	Mouth,Jaw,Teeth	**any**
**027**	Contusion	**36**	Multiple site	**any**

TBI codes, diagnostic imaging codes

ID	SNOMED CT Concept-ID	SNOMED CT FSN	Short Code	Recommended substitute procedure	Preferred representation display term (Max string length = 40)	Synonym (Max string length = 40)	Synonym 2 (Max string length = 40)	Synonym 3 (Max string length = 40)	Include?
58318	432814005	Computed tomography of base of skull (procedure)	CBSSK	0	CT Base of skull	Base of skull CT	Skull base CT	0	Include
58319	431619004	Computed tomography of base of skull with contrast (procedure)	CBSSKC	0	CT Base of skull with contrast	Base of skull CT with contrast	Skull base CT with contrast	0	Include
63583	1110811000000104	Computed tomography of bilateral temporal bones, brain and neck with contrast (procedure)	CBNTBC	0	CT Brain neck temporal bones Cont Both	0	0	0	Include
369	408754009	Computed tomography of entire head (procedure)	CSKUH	0	CT Head	Head CT	0	0	Include
62828	429858000	Computed tomography of head and neck (procedure)	CSKNE	0	CT Head and neck	0	0	0	Include
59389	429858000	Computed tomography of head and neck (procedure)	CSKNE	CSKNE	CT Head and neck	0	0	0	Include

61260	448760006	Computed tomography of head and neck and thorax and abdomen with contrast (procedure)	CHNTAC	0	CT Head neck thorax abdomen contrast	CT Head neck chest abdomen contrast	0	0	Include
61230	448335009	Computed tomography of head and neck with contrast (procedure)	CHENEC	0	CT Head and neck with contrast	0	0	0	Include
61075	777281000000109	Computed tomography of head and neck with contrast (procedure)	CHENEC	CHENE C	CT Head and neck with contrast	0	0	0	Include
63032	702762001	Computed tomography of head and orbits (procedure)	CORBH	0	CT Head and orbits	0	0	0	Include
63466	702763006	Computed tomography of head and orbits with contrast (procedure)	CORBHC	0	CT Head and orbits with contrast	0	0	0	Include
60912	444708006	Computed tomography of head and thorax (procedure)	CHTH	0	CT Head and thorax	0	0	0	Include
60634	523341000000100	Computed tomography of head and thorax (procedure)	CHTH	CHTH	CT Head and thorax	0	0	0	Include
62362	444709003	Computed tomography of head and thorax with contrast (procedure)	CHTHC	0	CT Head and thorax with contrast	0	0	0	Include
60913	444709003	Computed tomography of head and thorax with contrast (procedure)	CHTHC	CHTHC	CT Head and thorax with contast	0	0	0	Include
60632	523371000000106	Computed tomography of head and thorax with contrast (procedure)	CHTHC	CHTHC	CT Head and thorax with contast	0	0	0	Include

60903	444618009	Computed tomography of head, abdomen and pelvis (procedure)	CHAP	0	CT Head abdomen and pelvis	0	0	0	Include
60633	523091000000108	Computed tomography of head, abdomen and pelvis (procedure)	CHAP	CHAP	CT Head abdomen and pelvis	0	0	0	Include
60904	444628000	Computed tomography of head, abdomen and pelvis with contrast (procedure)	CHAPC	0	CT Head abdomen pelvis with contrast	0	0	0	Include
60631	523141000000102	Computed tomography of head, abdomen and pelvis with contrast (procedure)	CHAPC	CHAPC	CT Head abdomen pelvis with contrast	0	0	0	Include
62678	715588003	Computed tomography of head, neck, abdomen and pelvis (procedure)	CHNAP	0	CT Head neck abdomen and pelvis	0	0	0	Include
62432	974801000000101	Computed tomography of head, neck, abdomen and pelvis (procedure)	CHNAP	CHNAP	CT Head neck abdomen and pelvis	0	0	0	Include
62929	719898006	Computed tomography of head, neck, abdomen and pelvis with contrast (procedure)	CHNAPC	0	CT Head neck Abdo pelvis with contrast	0	0	0	Include
62433	974811000000104	Computed tomography of head, neck, abdomen and pelvis with contrast (procedure)	CHNAPC	CHNAP C	CT Head neck Abdo pelvis with contrast	0	0	0	Include

62378	711278009	Computed tomography of head, neck, thorax and abdomen (procedure)	CHNTA	0	CT Head neck thorax and abdomen	0	0	0	Include
61440	810081000000106	Computed tomography of head, neck, thorax and abdomen (procedure)	CHNTA	CHNTA	CT Head neck thorax and abdomen	0	0	0	Include
61076	777291000000106	Computed tomography of head, neck, thorax and abdomen with contrast (procedure)	CHNTAC	CHNTA C	CT Head neck thorax abdomen contrast	CT Head neck chest abdomen contrast	0	0	Include
58254	354821000000100	Computed tomography of head, neck, thorax, abdomen and pelvis (procedure)	CBNTA	CBNTA	CT brain & neck & thorax & Abdo & pelvis	0	0	0	Include
60635	354821000000100	Computed tomography of head, neck, thorax, abdomen and pelvis (procedure)	CHNTAP	CHNTA P	CT Head neck thorax abdomen and pelvis	0	0	0	Include
60366	440331001	Computed tomography of head, neck, thorax, abdomen and pelvis (procedure)	CBNTA	0	CT Brain neck thorax Abdo and pelvis	0	0	0	Include
60673	440331001	Computed tomography of head, neck, thorax, abdomen and pelvis (procedure)	CHNTAP	0	CT Head neck thorax abdomen and pelvis	0	0	0	Include
61211	444630003	Computed tomography of head, neck, thorax, abdomen and pelvis with contrast (procedure)	CHNTPC	0	CT Head neck thorax Abdo and pelvis Cont	0	0	0	Include

60905	444633001	Computed tomography of head, thorax and abdomen (procedure)	CHTA	0	CT Head thorax and abdomen	0	0	0	Include
60637	523351000000102	Computed tomography of head, thorax and abdomen (procedure)	CHTA	CHTA	CT Head thorax and Abdomen	0	0	0	Include
60915	444758004	Computed tomography of head, thorax and abdomen with contrast (procedure)	CHTHAC	0	CT Head thorax abdomen with contrast	0	0	0	Include
60636	523171000000108	Computed tomography of head, thorax and abdomen with contrast (procedure)	CHTHAC	CHTHA C	CT Head thorax abdomen with contrast	0	0	0	Include
60929	445583005	Computed tomography of head, thorax, abdomen and pelvis (procedure)	CHTAP	0	CT Head thorax abdomen and pelvis	0	0	0	Include
60629	523361000000104	Computed tomography of head, thorax, abdomen and pelvis (procedure)	CHTAP	CHTAP	CT Head thorax abdomen and pelvis	0	0	0	Include
60908	444674001	Computed tomography of head, thorax, abdomen and pelvis with contrast (procedure)	CHTAPC	0	CT Head thorax Abdo pelvis with contrast	0	0	0	Include
60630	523161000000101	Computed tomography of head, thorax, abdomen and pelvis with contrast (procedure)	CHTAPC	CHTAP C	CT Head thorax Abdo pelvis with contrast	0	0	0	Include

55699	310081000000109	Computerised tomography of base of skull (procedure)	CBSSK	CBSSK	CT Base of skull	Base of skull CT	Skull base CT	0	Include
55700	313151000000109	Computerised tomography of base of skull with contrast (procedure)	CBSSKC	CBSSKC	CT Base of skull with contrast	Base of skull CT with contrast	Skull base CT with contrast	0	Include
57041	316241000000107	Computerised tomography of head and neck (procedure)	CSKNE	CSKNE	CT Head and neck	0	0	0	Include
55804	396207002	Computerized axial tomography of brain with radiopaque contrast (procedure)	CSKUHC	0	CT Head with contrast	Head CT with contrast	0	0	Include
57	408754009	CT of head	CBSSK	CBSSK	CT Base of skull	Base of skull CT	Skull base CT	0	Include
58	408754009	CT of head	CBSSKC	CBSSKC	CT Base of skull with contrast	Base of skull CT with contrast	Skull base CT with contrast	0	Include
3189	408754009	CT of head	CSKNE	CSKNE	CT Head and neck	0	0	0	Include
373	408754009	CT of head	CSKWC	0	CT Head with & without contrast	0	0	0	Include
368	408754009	CT of head	CSKUC	CSKUH C	CT Head With Contrast	0	0	0	Include
372	408754009	CT of head	CSKUHC	CSKUH C	CT Head with contrast	Head CT with contrast	0	0	Include

378	408754009	CT of head	CTEMP	CTEMP	CT Temporal bones	Temporal bone CT	0	0	Include
394	408754009	CT of head	CTMJB	CTMJB	CT Temporomandibular joint Both	Temporomandibular joint CT Both	TMJ CT	0	Include
330	408754009	CT of head	CRTSKR	CRTSKR	CT Radiotherapy planning scan head	Head radiotherapy planning scan CT	Planning scan head	Radiotherapy planning head CT	Include
331	408754009	CT of head	CRTSMR	CRTSMR	CT Radiotherapy planning scan SMART	SMART radiotherapy planning scan CT	Planning scan SMART	Radiotherapy planning SMART CT	Include
367	408754009	CT of head	CSKPE	CSKPE	CT Brain perfusion study	Brain perfusion CT	0	0	Include
89	408754009	CT of head	CCISTT	CCISTT	CT Cisternography	Cisternography CT	0	0	Include
94	408754009	CT of head	CCPP	CCPP	CT Cranioplasty planning	Cranioplasty planning CT	0	0	Include
101	408754009	CT of head	CDCGB	CDCGB	CT Dacrocystogram Both	Dacrocystogram CT Both	0	0	Include

102	408754009	CT of head	CDCGL	CDCGL	CT Dacrocystogram Lt	Dacrocystogram CT Lt	0	0	Include
103	408754009	CT of head	CDCGR	CDCGR	CT Dacrocystogram Rt	Dacrocystogram CT Rt	0	0	Include
3066	42075002	Diagnostic radiography of skull (procedure)	XSKUH	0	XR Skull	Skull XR	SXR	0	Maybe
371	363680008	radiographic imaging procedure	CSKUHB	CSKUHB	CT Guided brain biopsy	Brain biopsy CT guided	Biopsy brain CT guided	0	Maybe
2657	363680008	radiographic imaging procedure	USKUH	USKUH	US Skull	Skull US	0	0	Maybe
2718	363680008	radiographic imaging procedure	UTRCD	UTRCD	US Transcranial doppler	Transcranial doppler US	0	0	Maybe
919	42075002	X-ray of skull	FSKUB	FSKUH	Fluoroscopy skull	Skull fluoroscopy	0	0	Maybe
921	42075002	X-ray of skull	FSKUH	FSKUH	Fluoroscopy skull	Skull fluoroscopy	0	0	Maybe
920	42075002	X-ray of skull	FSKUBM	FSKUHM	Mobile image intensifier skull	Skull mobile II	II skull	0	Maybe
922	42075002	X-ray of skull	FSKUHM	FSKUHM	Mobile image intensifier skull	Skull mobile image intensifier	Image intensifier skull	0	Maybe
3065	42075002	X-ray of skull	XSKUB	XSKUH	XR Skull	Skull XR	SXR	0	Maybe

57948	303678006	computed tomography of regions (procedure)	CBNTA	CBNTA	CT brain & neck & thorax & Abdo & pelvis	0	0	0	Maybe
