# Supplementary material for: Predictors of persisting symptoms after concussion in children following a traumatic brain injury: a longitudinal retrospective cohort study
Source: BMJ Paediatr Open. 2025 Apr 5;9(1):e003036. doi: 10.1136/bmjpo-2024-003036 (PMC11973773; doi:10.1136/bmjpo-2024-003036)

# Supplementary materials

| Table of contents |
| --- |
| Table S1: Sample descriptives by sensitivity PSaC outcome (PSaC or PCS diagnosis code or two or more symptoms 3-12 months after TBI)  Table S2: Sample descriptives by sensitivity PSaC outcome (PSaC or PCS diagnosis code or one or more symptom 3 months-3 years after TBI)  Table S3: Sample descriptives by sensitivity PSaC outcome (PSaC or PCS diagnosis code or two or more symptoms 3 months-3 years after TBI)  Table S4: Sample descriptives by sensitivity PSaC outcome (PSaC or PCS diagnosis code or one or more symptoms (excluding headaches, depression and sleep disorders) 3-12 months after TBI))  Table S5: Multivariable logistic regression models for each of the sensitivity PSaC outcomes  Table S6: Multivariable logistic regression model stratified by age group with primary PSaC outcome (PSaC or PCS diagnosis code or one or more symptoms 3-12 months after TBI)  Table S7: Multivariable logistic regression models including primary PSaC outcome and interaction terms  Table S8: Multivariable logistic regression, LASSO, elastic net, ridge regression and backwards selection logistic regression models including primary PSaC outcome  Figure S1: Full logistic regression model calibration plot  Figure S2: Backwards selection regression model calibration plot  Figure S3: Bootstrap internal validation calibration plot |

Table S1: Sample descriptives by sensitivity PSaC outcome (PSaC or PCS diagnosis code or two or more symptoms 3-12 months after TBI)

|  | PSaC or suspected PSaC, N (%) | | | |
| --- | --- | --- | --- | --- |
|  | No | Yes | Total |  |
| N | 137,246 (99.5%) | 627 (0.5%) | 137,873 |  |
| Age (years) at time of TBI, mean (SD) | 6.50 (5.48) | 12.44 (5.73) | 6.52 (5.50) | <0.001 |
| Sex |  |  |  |  |
| Male | 85,454 (62.3%) | 356 (56.8%) | 85,810 (62.2%) | 0.018 |
| Female | 51,788 (37.7%) | 271 (43.2%) | 52,059 (37.8%) |  |
| Indeterminate | - (<0.1%) | - (<0.1%) | - (<0.1%) |  |
| Deprivation quintile |  |  |  |  |
| 1 (least deprived) | 29,700 (21.7%) | 150 (23.9%) | 29,850 (21.7%) | 0.149 |
| 2 | 25,840 (18.8%) | 108 (17.2%) | 25,948 (18.8%) |  |
| 3 | 24,478 (17.8%) | 111 (17.7%) | 24,589 (17.8%) |  |
| 4 | 26,373 (19.2%) | 136 (21.7%) | 26,509 (19.2%) |  |
| 5 (most deprived) | 30,749 (22.4%) | 122 (19.5%) | 30,871 (22.4%) |  |
| Ethnicity |  |  |  |  |
| Asian | 7,827 (6.1%) | 23 (3.9%) | 7,850 (6.1%) | 0.026 |
| Black | 4,693 (3.7%) | 13 (2.2%) | 4,706 (3.6%) |  |
| Mixed | 4,773 (3.7%) | 26 (4.4%) | 4,799 (3.7%) |  |
| Other | 2,396 (1.9%) | 12 (2.0%) | 2,408 (1.9%) |  |
| White | 105,633 (82.2%) | 507 (86.1%) | 106,140 (82.2%) |  |
| Unknown | 3,148 (2.5%) | 8 (1.4%) | 3,156 (2.4%) |  |
| History of headaches before TBI |  |  |  |  |
| No | 134,013 (97.6%) | 493 (78.6%) | 134,506 (97.6%) | <0.001 |
| Yes | 3,233 (2.4%) | 134 (21.4%) | 3,367 (2.4%) |  |
| History of learning disability before TBI |  |  |  |  |
| No | 135,965 (99.1%) | 608 (97.0%) | 136,573 (99.1%) | <0.001 |
| Yes | 1,281 (0.9%) | 19 (3.0%) | 1,300 (0.9%) |  |
| History of ADHD before TBI |  |  |  |  |
| No | 136,486 (99.4%) | 611 (97.4%) | 137,097 (99.4%) | <0.001 |
| Yes | 760 (0.6%) | 16 (2.6%) | 776 (0.6%) |  |
| History of anxiety before TBI |  |  |  |  |
| No | 135,796 (98.9%) | 563 (89.8%) | 136,359 (98.9%) | <0.001 |
| Yes | 1,450 (1.1%) | 64 (10.2%) | 1,514 (1.1%) |  |
| History of depression before TBI |  |  |  |  |
| No | 136,537 (99.5%) | 565 (90.1%) | 137,102 (99.4%) | <0.001 |
| Yes | 709 (0.5%) | 62 (9.9%) | 771 (0.6%) |  |
| History of sleep disorder before TBI |  |  |  |  |
| No | 135,432 (98.7%) | 594 (94.7%) | 136,026 (98.7%) | <0.001 |
| Yes | 1,814 (1.3%) | 33 (5.3%) | 1,847 (1.3%) |  |

Table S2: Sample descriptives by sensitivity PSaC outcome (PSaC or PCS diagnosis code or one or more symptom 3 months-3 years after TBI)

|  | PSaC or suspected PSaC | | | |
| --- | --- | --- | --- | --- |
|  | No | Yes | Total | P value |
| N | 126,374 (91.7) | 11,499 (8.3%) | 137,873 |  |
| Age (years) at time of TBI | 6.40 (5.41) | 7.87 (6.29) | 6.52 (5.50) | <0.001 |
| Sex |  |  |  |  |
| Male | 79,151 (62.6%) | 6,659 (57.9%) | 85,810 (62.2%) | <0.001 |
| Female | 47,219 (37.4%) | 4,840 (42.1%) | 52,059 (37.8%) |  |
| Indeterminate | - (<0.1%) | - (<0.1%) | - (<0.1%) |  |
| Deprivation quintile |  |  |  |  |
| 1 (least deprived) | 27,496 (21.8%) | 2,354 (20.5%) | 29,850 (21.7%) | <0.001 |
| 2 | 23,960 (19.0%) | 1,988 (17.3%) | 25,948 (18.8%) |  |
| 3 | 22,519 (17.8%) | 2,070 (18.0%) | 24,589 (17.8%) |  |
| 4 | 24,198 (19.2%) | 2,311 (20.1%) | 26,509 (19.2%) |  |
| 5 (most deprived) | 28,102 (22.3%) | 2,769 (24.1%) | 30,871 (22.4%) |  |
| Ethnicity |  |  |  |  |
| Asian | 6,989 (5.9%) | 861 (7.8%) | 7,850 (6.1%) | <0.001 |
| Black | 4,265 (3.6%) | 441 (4.0%) | 4,706 (3.6%) |  |
| Mixed | 4,363 (3.7%) | 436 (4.0%) | 4,799 (3.7%) |  |
| Other | 2,189 (1.9%) | 219 (2.0%) | 2,408 (1.9%) |  |
| White | 97,279 (82.4%) | 8,861 (80.7%) | 106,140 (82.2%) |  |
| Unknown | 3,000 (2.5%) | 156 (1.4%) | 3,156 (2.4%) |  |
| History of headaches before TBI |  |  |  |  |
| No | 124,088 (98.2%) | 10,418 (90.6%) | 134,506 (97.6%) | <0.001 |
| Yes | 2,286 (1.8%) | 1,081 (9.4%) | 3,367 (2.4%) |  |
| History of learning disability before TBI |  |  |  |  |
| No | 125,395 (99.2%) | 11,178 (97.2%) | 136,573 (99.1%) | <0.001 |
| Yes | 979 (0.8%) | 321 (2.8%) | 1,300 (0.9%) |  |
| History of ADHD before TBI |  |  |  |  |
| No | 125,850 (99.6%) | 11,247 (97.8%) | 137,097 (99.4%) | <0.001 |
| Yes | 524 (0.4%) | 252 (2.2%) | 776 (0.6%) |  |
| History of anxiety before TBI |  |  |  |  |
| No | 125,379 (99.2%) | 10,980 (95.5%) | 136,359 (98.9%) | <0.001 |
| Yes | 995 (0.8%) | 519 (4.5%) | 1,514 (1.1%) |  |
| History of depression before TBI |  |  |  |  |
| No | 126,008 (99.7%) | 11,094 (96.5%) | 137,102 (99.4%) | <0.001 |
| Yes | 366 (0.3%) | 405 (3.5%) | 771 (0.6%) |  |
| History of sleep disorder before TBI |  |  |  |  |
| No | 124,942 (98.9%) | 11,084 (96.4%) | 136,026 (98.7%) | <0.001 |
| Yes | 1,432 (1.1%) | 415 (3.6%) | 1,847 (1.3%) |  |

Table S3: Sample descriptives by sensitivity PSaC outcome (PSaC or PCS diagnosis code or two or more symptoms 3 months-3 years after TBI)

|  | PSaC or suspected PSaC | | | |
| --- | --- | --- | --- | --- |
|  | No | Yes | Total | P value |
| N | 135,702 (98.4%) | 2,171 (1.6%) | 137,873 |  |
| Age (years) at time of TBI | 6.45 (5.46) | 11.30 (5.97) | 6.52 (5.50) | <0.001 |
| Sex |  |  |  |  |
| Male | 84,632 (62.4%) | 1,178 (54.3%) | 85,810 (62.2%) | <0.001 |
| Female | 51,066 (37.6%) | 993 (45.7%) | 52,059 (37.8%) |  |
| Indeterminate | - (<0.1%) | - (<0.1%) | - (<0.1%) |  |
| Deprivation quintile |  |  |  |  |
| 1 (least deprived) | 29,402 (21.7%) | 448 (20.6%) | 29,850 (21.7%) | 0.110 |
| 2 | 25,573 (18.9%) | 375 (17.3%) | 25,948 (18.8%) |  |
| 3 | 24,196 (17.8%) | 393 (18.1%) | 24,589 (17.8%) |  |
| 4 | 26,056 (19.2%) | 453 (20.9%) | 26,509 (19.2%) |  |
| 5 (most deprived) | 30,369 (22.4%) | 502 (23.1%) | 30,871 (22.4%) |  |
| Ethnicity |  |  |  |  |
| Asian | 7,720 (6.1%) | 130 (6.3%) | 7,850 (6.1%) | <0.001 |
| Black | 4,648 (3.7%) | 58 (2.8%) | 4,706 (3.6%) |  |
| Mixed | 4,727 (3.7%) | 72 (3.5%) | 4,799 (3.7%) |  |
| Other | 2,376 (1.9%) | 32 (1.6%) | 2,408 (1.9%) |  |
| White | 104,400 (82.2%) | 1,740 (84.7%) | 106,140 (82.2%) |  |
| Unknown | 3,133 (2.5%) | 23 (1.1%) | 3,156 (2.4%) |  |
| History of headaches before TBI |  |  |  |  |
| No | 132,704 (97.8%) | 1,802 (83.0%) | 134,506 (97.6%) | <0.001 |
| Yes | 2,998 (2.2%) | 369 (17.0%) | 3,367 (2.4%) |  |
| History of learning disability before TBI |  |  |  |  |
| No | 134,495 (99.1%) | 2,078 (95.7%) | 136,573 (99.1%) | <0.001 |
| Yes | 1,207 (0.9%) | 93 (4.3%) | 1,300 (0.9%) |  |
| History of ADHD before TBI |  |  |  |  |
| No | 134,986 (99.5%) | 2,111 (97.2%) | 137,097 (99.4%) | <0.001 |
| Yes | 716 (0.5%) | 60 (2.8%) | 776 (0.6%) |  |
| History of anxiety before TBI |  |  |  |  |
| No | 134,373 (99.0%) | 1,986 (91.5%) | 136,359 (98.9%) | <0.001 |
| Yes | 1,329 (1.0%) | 185 (8.5%) | 1,514 (1.1%) |  |
| History of depression before TBI |  |  |  |  |
| No | 135,103 (99.6%) | 1,999 (92.1%) | 137,102 (99.4%) | <0.001 |
| Yes | 599 (0.4%) | 172 (7.9%) | 771 (0.6%) |  |
| History of sleep disorder before TBI |  |  |  |  |
| No | 133,970 (98.7%) | 2,056 (94.7%) | 136,026 (98.7%) | <0.001 |
| Yes | 1,732 (1.3%) | 115 (5.3%) | 1,847 (1.3%) |  |

Table S4: Sample descriptives by sensitivity PSaC outcome (PSaC or PCS diagnosis code or one or more symptoms (excluding headaches, depression and sleep disorders) 3-12 months after TBI))

|  | PSaC or suspected PSaC | | | |
| --- | --- | --- | --- | --- |
|  | No | Yes | Total | P value |
| N | 134,540 (97.6%) | 3,333 (2.4%) | 137,873 |  |
| Age (years) at time of TBI | 6.52 (5.48) | 6.72 (6.26) | 6.52 (5.50) | 0.034 |
| Sex |  |  |  |  |
| Male | 83,872 (62.3%) | 1,938 (58.1%) | 85,810 (62.2%) | <0.001 |
| Female | 50,664 (37.7%) | 1,395 (41.9%) | 52,059 (37.8%) |  |
| Indeterminate | - (<0.1%) | - (<0.1%) | - (<0.1%) |  |
| Deprivation quintile |  |  |  |  |
| 1 (least deprived) | 29,164 (21.7%) | 686 (20.6%) | 29,850 (21.7%) | 0.002 |
| 2 | 25,387 (18.9%) | 561 (16.8%) | 25,948 (18.8%) |  |
| 3 | 23,982 (17.8%) | 607 (18.2%) | 24,589 (17.8%) |  |
| 4 | 25,848 (19.2%) | 661 (19.8%) | 26,509 (19.2%) |  |
| 5 (most deprived) | 30,054 (22.4%) | 817 (24.5%) | 30,871 (22.4%) |  |
| Ethnicity |  |  |  |  |
| Asian | 7,567 (6.0%) | 283 (8.9%) | 7,850 (6.1%) | <0.001 |
| Black | 4,579 (3.6%) | 127 (4.0%) | 4,706 (3.6%) |  |
| Mixed | 4,660 (3.7%) | 139 (4.4%) | 4,799 (3.7%) |  |
| Other | 2,341 (1.9%) | 67 (2.1%) | 2,408 (1.9%) |  |
| White | 103,613 (82.3%) | 2,527 (79.4%) | 106,140 (82.2%) |  |
| Unknown | 3,115 (2.5%) | 41 (1.3%) | 3,156 (2.4%) |  |
| History of headaches before TBI |  |  |  |  |
| No | 131,524 (97.8%) | 3,060 (91.8%) | 134,584 (97.6%) | <0.001 |
| Yes | 3,016 (2.2%) | 273 (8.2%) | 3,289 (2.4%) |  |
| History of learning disability before TBI |  |  |  |  |
| No | 133,328 (99.1%) | 3,245 (97.4%) | 136,573 (99.1%) | <0.001 |
| Yes | 1,212 (0.9%) | 88 (2.6%) | 1,300 (0.9%) |  |
| History of ADHD before TBI |  |  |  |  |
| No | 133,855 (99.5%) | 3,242 (97.3%) | 137,097 (99.4%) | <0.001 |
| Yes | 685 (0.5%) | 91 (2.7%) | 776 (0.6%) |  |
| History of anxiety before TBI |  |  |  |  |
| No | 133,177 (99.0%) | 3,182 (95.5%) | 136,359 (98.9%) | <0.001 |
| Yes | 1,363 (1.0%) | 151 (4.5%) | 1,514 (1.1%) |  |
| History of depression before TBI |  |  |  |  |
| No | 133,876 (99.5%) | 3,226 (96.8%) | 137,102 (99.4%) | <0.001 |
| Yes | 664 (0.5%) | 107 (3.2%) | 771 (0.6%) |  |
| History of sleep disorder before TBI |  |  |  |  |
| No | 132,799 (98.7%) | 3,227 (96.8%) | 136,026 (98.7%) | <0.001 |
| Yes | 1,741 (1.3%) | 106 (3.2%) | 1,847 (1.3%) |  |

Table S5: Multivariable logistic regression models for each of the sensitivity PSaC outcomes

| N=128966 | PSaC or PCS diagnosis code or two or more symptoms 3-12 months after TBI | PSaC or PCS diagnosis code or one or more symptoms 3 months-3 years after TBI | PSaC or PCS diagnosis code or two or more symptoms 3 months-3 years after TBI | PSaC or PCS diagnosis code or one or more symptom (excluding headaches, depression, sleep disorders) 3-12 months after TBI |
| --- | --- | --- | --- | --- |
|  | **OR [95% CI], P value** | | | |
| Age (years) at time of TBI | 1.15 [1.13- 1.17], <0.001 | 1.03 [1.02- 1.03], <0.001 | 1.12 [1.11- 1.13], <0.001 | 0.98 [0.98- 0.99], <0.001 |
| Sex (compared with male) |  |  |  |  |
| Female | 1.17 [0.99- 1.39], 0.072 | 1.22 [1.17- 1.27], <0.001 | 1.38 [1.26- 1.51], <0.001 | 1.14 [1.06- 1.23], <0.001 |
| Deprivation quintile (compared with 1= least deprived) |  |  |  |  |
| 2 | 0.89 [0.68- 1.16], 0.382 | 0.98 [0.92- 1.05], 0.632 | 1.04 [0.90- 1.21], 0.566 | 0.95 [0.85- 1.07], 0.430 |
| 3 | 1.01 [0.78- 1.31], 0.938 | 1.09 [1.02- 1.16], 0.010 | 1.19 [1.03- 1.38], 0.021 | 1.09 [0.97- 1.22], 0.137 |
| 4 | 1.09 [0.85- 1.40], 0.488 | 1.10 [1.03- 1.17], 0.004 | 1.23 [1.07- 1.42], 0.004 | 1.06 [0.95- 1.19], 0.325 |
| 5 (most deprived) | 0.92 [0.72- 1.19], 0.537 | 1.14 [1.08- 1.22], <0.001 | 1.24 [1.08- 1.42], 0.003 | 1.13 [1.01- 1.26], 0.030 |
| Ethnicity (compared with white) |  |  |  |  |
| Asian | 0.75 [0.49- 1.14], 0.182 | 1.40 [1.30- 1.51], <0.001 | 1.16 [0.96- 1.39], 0.125 | 1.54 [1.36- 1.75], <0.001 |
| Black | 0.61 [0.35- 1.07], 0.086 | 1.08 [0.98- 1.20], 0.130 | 0.73 [0.55- 0.95], 0.020 | 1.10 [0.91- 1.32], 0.314 |
| Mixed | 1.51 [1.01- 2.26], 0.042 | 1.15 [1.04- 1.28], 0.007 | 1.12 [0.88- 1.43], 0.346 | 1.22 [1.02- 1.45], 0.028 |
| Other | 1.19 [0.66- 2.12], 0.567 | 1.13 [0.98- 1.31], 0.084 | 0.88 [0.61- 1.26], 0.477 | 1.19 [0.93- 1.52], 0.173 |
| Unknown | 0.49 [0.24- 1.00], 0.049 | 0.57 [0.48- 0.67], <0.001 | 0.42 [0.27- 0.63], <0.001 | 0.57 [0.42- 0.78], <0.001 |
| History of headaches before TBI | 3.57 [2.85- 4.48], <0.001 | 3.55 [3.25- 3.87], <0.001 | 3.18 [2.77- 3.65], <0.001 | 3.02 [2.59- 3.51], <0.001 |
| History of learning disability before TBI | 1.44 [0.88- 2.38], 0.149 | 2.34 [2.03- 2.70], <0.001 | 2.47 [1.94- 3.14], <0.001 | 1.89 [1.49- 2.41], <0.001 |
| History of ADHD before TBI | 1.22 [0.70- 2.11], 0.488 | 2.81 [2.37- 3.34], <0.001 | 1.42 [1.05- 1.93], 0.023 | 3.73 [2.91- 4.79], <0.001 |
| History of anxiety before TBI | 2.01 [1.44- 2.80], <0.001 | 2.71 [2.38- 3.09], <0.001 | 2.18 [1.78- 2.67], <0.001 | 2.66 [2.17- 3.27], <0.001 |
| History of depression before TBI | 2.39 [1.68- 3.39], <0.001 | 4.68 [3.95- 5.55], <0.001 | 2.81 [2.26- 3.50], <0.001 | 2.85 [2.19- 3.71], <0.001 |
| History of sleep disorder before TBI | 1.77 [1.19- 2.65], 0.005 | 2.26 [2.00- 2.56], <0.001 | 2.23 [1.79- 2.78], <0.001 | 1.69 [1.36- 2.10], <0.001 |

Table S6: Multivariable logistic regression model stratified by age group with primary PSaC outcome (PSaC or PCS diagnosis code or one or more symptoms 3-12 months after TBI)

|  | Age <5 years  (N=69,035) | | | Age ≥5 years  (N=59,927) |
| --- | --- | --- | --- | --- |
|  | **OR [95% CI], P value** | | | |
| Age (years) at time of TBI | | 0.84 [0.82- 0.87], <0.001 | 1.08 [1.07- 1.09], <0.001 | |
| Sex (compared with male) | |  |  | |
| Female | | 0.97 [0.89- 1.06], 0.463 | 1.43 [1.31- 1.56], <0.001 | |
| Deprivation quintile (compared with 1= least deprived) | |  |  | |
| 2 | | 1.02 [0.88- 1.18], 0.787 | 0.88 [0.77- 1.01], 0.080 | |
| 3 | | 1.11 [0.96- 1.28], 0.164 | 1.03 [0.90- 1.18], 0.686 | |
| 4 | | 1.09 [0.94- 1.25], 0.245 | 1.01 [0.88- 1.15], 0.898 | |
| 5 (most deprived) | | 1.18 [1.03- 1.35], 0.015 | 0.95 [0.83- 1.09], 0.461 | |
| Ethnicity (compared with white) | |  |  | |
| Asian | | 1.58 [1.36- 1.85], <0.001 | 1.29 [1.08- 1.54], 0.005 | |
| Black | | 1.35 [1.08- 1.68], 0.007 | 1.03 [0.82- 1.29], 0.828 | |
| Mixed | | 1.11 [0.90- 1.36], 0.342 | 1.30 [1.02- 1.65], 0.032 | |
| Other | | 1.24 [0.91- 1.68], 0.170 | 1.26 [0.93- 1.71], 0.138 | |
| Unknown | | 0.59 [0.39- 0.89], 0.013 | 0.58 [0.42- 0.81], 0.001 | |
| History of headaches* | | 4.21 [2.29- 7.73], <0.001 | 3.47 [3.08- 3.91], <0.001 | |
| History of learning disability* | | 4.24 [2.95- 6.09], <0.001 | 1.80 [1.41- 2.29], <0.001 | |
| History of ADHD* | | 1.51 [0.16- 14.31], 0.721 | 2.63 [2.08- 3.33], <0.001 | |
| History of anxiety* | | 5.96 [4.04- 8.79], <0.001 | 2.13 [1.77- 2.56], <0.001 | |
| History of depression* | | - | 3.21 [2.62- 3.92], <0.001 | |
| History of sleep disorder* | | 4.71 [3.56- 6.24], <0.001 | 2.07 [1.69- 2.54], <0.001 | |

Table S7: Multivariable logistic regression models including primary PSaC outcome (PSaC or PCS diagnosis code or one or more symptom 3-12 months after TBI) and interaction terms

| N=128966 | | OR (95% CI), p | | | | | |
| --- | --- | --- | --- | --- | --- | --- | --- |
| Interaction terms included | No interactions | | Age and learning disability | Age and anxiety | Age and sleep disorder | Sex and depression | All interactions |
| Age (years) at time of TBI | 1.02 [1.01- 1.03], <0.001 | | 1.02 [1.01- 1.03], <0.001 | 1.02 [1.01- 1.03], <0.001 | 1.02 [1.01- 1.03], <0.001 | 1.02 [1.01- 1.02], <0.001 | 1.02 [1.02- 1.03], <0.001 |
| Sex (compared with male) |  | |  |  |  |  |  |
| Female | 1.20 [1.13- 1.28], <0.001 | | 1.20 [1.13- 1.28], <0.001 | 1.20 [1.13- 1.28], <0.001 | 1.20 [1.13- 1.28], <0.001 | 1.18 [1.11- 1.26], <0.001 | 1.19 [1.11- 1.26], <0.001 |
| Deprivation quintile (compared with 1= least deprived) |  | |  |  |  |  |  |
| 2 | 0.95 [0.86- 1.05], 0.345 | | 0.95 [0.86- 1.05], 0.354 | 0.95 [0.86- 1.05], 0.345 | 0.95 [0.86- 1.05], 0.347 | 0.95 [0.86- 1.05], 0.359 | 0.95 [0.86- 1.06], 0.368 |
| 3 | 1.08 [0.98- 1.19], 0.144 | | 1.08 [0.97- 1.19], 0.145 | 1.08 [0.97- 1.19], 0.148 | 1.08 [0.97- 1.19], 0.147 | 1.08 [0.97- 1.19], 0.145 | 1.07 [0.97- 1.19], 0.153 |
| 4 | 1.06 [0.96- 1.17], 0.232 | | 1.06 [0.96- 1.17], 0.231 | 1.06 [0.96- 1.17], 0.225 | 1.06 [0.96- 1.17], 0.229 | 1.06 [0.96- 1.17], 0.240 | 1.06 [0.96- 1.17], 0.230 |
| 5 (most deprived) | 1.07 [0.98- 1.18], 0.141 | | 1.07 [0.98- 1.18], 0.145 | 1.07 [0.98- 1.18], 0.132 | 1.07 [0.98- 1.18], 0.142 | 1.07 [0.98- 1.18], 0.148 | 1.07 [0.98- 1.18], 0.142 |
| Ethnicity (compared with white) |  | |  |  |  |  |  |
| Asian | 1.37 [1.22- 1.54], <0.001 | | 1.37 [1.22- 1.54], <0.001 | 1.37 [1.22- 1.54], <0.001 | 1.37 [1.22- 1.54], <0.001 | 1.37 [1.22- 1.54], <0.001 | 1.37 [1.22- 1.54], <0.001 |
| Black | 1.11 [0.95- 1.30], 0.199 | | 1.11 [0.95- 1.30], 0.205 | 1.11 [0.94- 1.30], 0.213 | 1.11 [0.94- 1.30], 0.211 | 1.11 [0.95- 1.30], 0.203 | 1.10 [0.94- 1.29], 0.232 |
| Mixed | 1.18 [1.01- 1.37], 0.041 | | 1.18 [1.01- 1.38], 0.040 | 1.18 [1.01- 1.38], 0.040 | 1.18 [1.01- 1.38], 0.040 | 1.17 [1.00- 1.37], 0.044 | 1.18 [1.01- 1.38], 0.041 |
| Other | 1.19 [0.96- 1.48], 0.112 | | 1.19 [0.96- 1.48], 0.115 | 1.19 [0.96- 1.48], 0.110 | 1.19 [0.96- 1.48], 0.111 | 1.20 [0.96- 1.48], 0.105 | 1.19 [0.96- 1.48], 0.106 |
| Unknown | 0.58 [0.45- 0.75], <0.001 | | 0.58 [0.45- 0.75], <0.001 | 0.58 [0.45- 0.75], <0.001 | 0.58 [0.45- 0.75], <0.001 | 0.58 [0.45- 0.75], <0.001 | 0.58 [0.45- 0.75], <0.001 |
| History of headaches before TBI | 3.52 [3.13- 3.95], <0.001 | | 3.51 [3.12- 3.94], <0.001 | 3.53 [3.14- 3.96], <0.001 | 3.54 [3.15- 3.98], <0.001 | 3.51 [3.13- 3.95], <0.001 | 3.54 [3.15- 3.98], <0.001 |
| History of learning disability before TBI | 2.06 [1.69- 2.52], <0.001 | | 3.40 [2.26- 5.10], <0.001 | 2.06 [1.69- 2.52], <0.001 | 2.06 [1.68- 2.51], <0.001 | 2.07 [1.70- 2.53], <0.001 | 3.10 [2.06- 4.68], <0.001 |
| History of ADHD before TBI | 2.41 [1.91- 3.04], <0.001 | | 2.51 [1.99- 3.16], <0.001 | 2.42 [1.92- 3.05], <0.001 | 2.45 [1.94- 3.09], <0.001 | 2.48 [1.97- 3.12], <0.001 | 2.60 [2.06- 3.27], <0.001 |
| History of anxiety before TBI | 2.58 [2.18- 3.05], <0.001 | | 2.59 [2.19- 3.07], <0.001 | 4.14 [2.75- 6.23], <0.001 | 2.59 [2.19- 3.07], <0.001 | 2.56 [2.16- 3.04], <0.001 | 4.11 [2.73- 6.18], <0.001 |
| History of depression before TBI | 4.00 [3.28- 4.89], <0.001 | | 4.02 [3.30- 4.91], <0.001 | 4.26 [3.47- 5.23], <0.001 | 4.08 [3.34- 4.98], <0.001 | 2.63 [1.85- 3.74], <0.001 | 2.94 [2.07- 4.17], <0.001 |
| History of sleep disorder before TBI | 2.35 [1.99- 2.78], <0.001 | | 2.34 [1.98- 2.76], <0.001 | 2.34 [1.98- 2.76], <0.001 | 3.78 [2.74- 5.20], <0.001 | 2.37 [2.01- 2.79], <0.001 | 3.55 [2.57- 4.92], <0.001 |
| *History of learning disability before TBI x Age at time of TBI* |  | | 0.95 [0.92- 0.99], 0.008 |  |  |  | 0.96 [0.93- 1.00], 0.034 |
| *History of anxiety before TBI x Age at time of TBI* |  | |  | 0.97 [0.94- 0.99], 0.015 |  |  | 0.97 [0.94- 0.99], 0.018 |
| *History of sleep disorder before TBI x Age at time of TBI* |  | |  |  | 0.95 [0.92- 0.98], 0.001 |  | 0.96 [0.93- 0.99], 0.005 |
| *History of depression before TBI x Female sex* |  | |  |  |  | 1.84 [1.23- 2.76], 0.003 | 1.77 [1.19- 2.64], 0.005 |

Table S8: Multivariable logistic regression, LASSO, elastic net, ridge regression and backwards selection logistic regression models including primary PSaC outcome (PSaC or PCS diagnosis code or one or more symptoms 3-12 months after TBI)

|  | Logistic regression  OR (95% CI), p | LASSO  OR | Elastic net  OR | Ridge regression  OR | Backwards selection (p<0.1)  OR (95% CI) |
| --- | --- | --- | --- | --- | --- |
| N=128966 |  |  |  |  |  |
| Age (years) at time of TBI | 1.02 [1.01- 1.03], <0.001 | 1.10 | 1.10 | 1.10 | 1.02 [1.01- 1.03], <0.001 |
| Sex |  |  |  |  |  |
| Male | 1.00 | 0.92 | 0.96 | 0.96 | 1.00 |
| Female | 1.20 [1.13- 1.28], <0.001 |  | 1.04 | 1.04 | 1.20 [1.12- 1.28], <0.001 |
| IMD |  |  |  |  |  |
| 1 (least deprived) | 1.00 | 0.98 | 0.98 | 0.99 | 1.00 |
| 2 | 0.95 [0.86- 1.05], 0.345 | 0.96 | 0.96 | 0.97 |  |
| 3 | 1.08 [0.98- 1.19], 0.144 | 1.00 | 1.00 | 1.02 | 1.10 [1.01- 1.20], 0.026 |
| 4 | 1.06 [0.96- 1.17], 0.232 |  |  | 1.01 | 1.09 [1.00- 1.19], 0.039 |
| 5 (most deprived) | 1.07 [0.98- 1.18], 0.141 | 1.00 | 1.00 | 1.02 | 1.11 [1.02- 1.20], 0.011 |
| Ethnicity |  |  |  |  |  |
| White | 1.00 |  |  |  | 1.00 |
| Asian | 1.37 [1.22- 1.54], <0.001 | 1.08 | 1.08 | 1.07 | 1.36 [1.21- 1.52], <0.001 |
| Black | 1.11 [0.95- 1.30], 0.199 | 1.02 | 1.02 | 1.02 |  |
| Mixed | 1.18 [1.01- 1.37], 0.041 | 1.03 | 1.03 | 1.03 | 1.16 [1.00- 1.36], 0.054 |
| Other | 1.19 [0.96- 1.48], 0.112 | 1.02 | 1.02 | 1.02 |  |
| Unknown | 0.58 [0.45- 0.75], <0.001 | 0.92 | 0.92 | 0.93 | 0.58 [0.44- 0.75], <0.001 |
| History of headaches before TBI | 3.52 [3.13- 3.95], <0.001 | 1.21 | 1.21 | 1.21 | 3.52 [3.13- 3.95], <0.001 |
| History of learning disability before TBI | 2.06 [1.69- 2.52], <0.001 | 1.07 | 1.07 | 1.07 | 2.06 [1.68- 2.52], <0.001 |
| History of ADHD before TBI | 2.41 [1.91- 3.04], <0.001 | 1.07 | 1.07 | 1.07 | 2.40 [1.90- 3.03], <0.001 |
| History of anxiety before TBI | 2.58 [2.18- 3.05], <0.001 | 1.10 | 1.10 | 1.10 | 2.57 [2.17- 3.05], <0.001 |
| History of depression before TBI | 4.00 [3.28- 4.89], <0.001 | 1.11 | 1.11 | 1.11 | 3.99 [3.27- 4.88], <0.001 |
| History of sleep disorder before TBI | 2.35 [1.99- 2.78], <0.001 | 1.11 | 1.10 | 1.10 | 2.36 [2.00- 2.78], <0.001 |
| C-statistic (CI) | 0.6163 [0.60678-0.62588] |  |  |  | 0.6140 [0.60437-0.62356] |

Figure S1: Full logistic regression model calibration plot


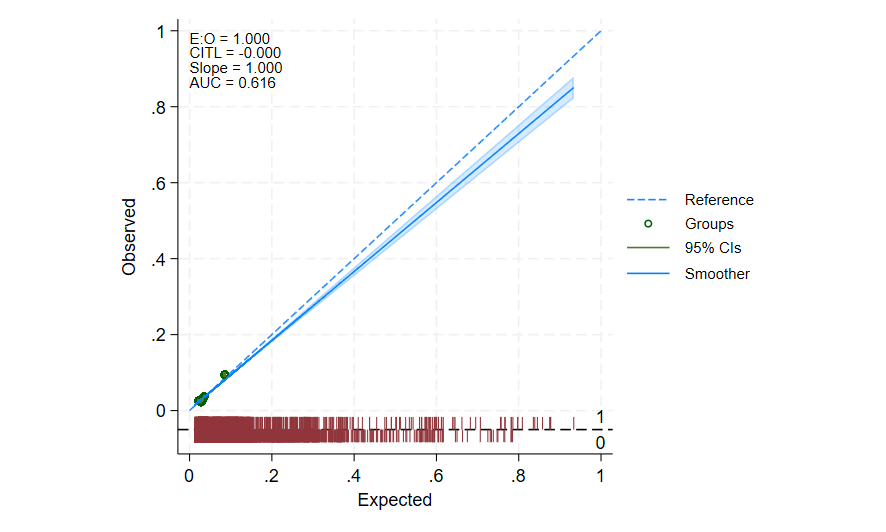


Figure S2: Backwards selection regression model calibration plot


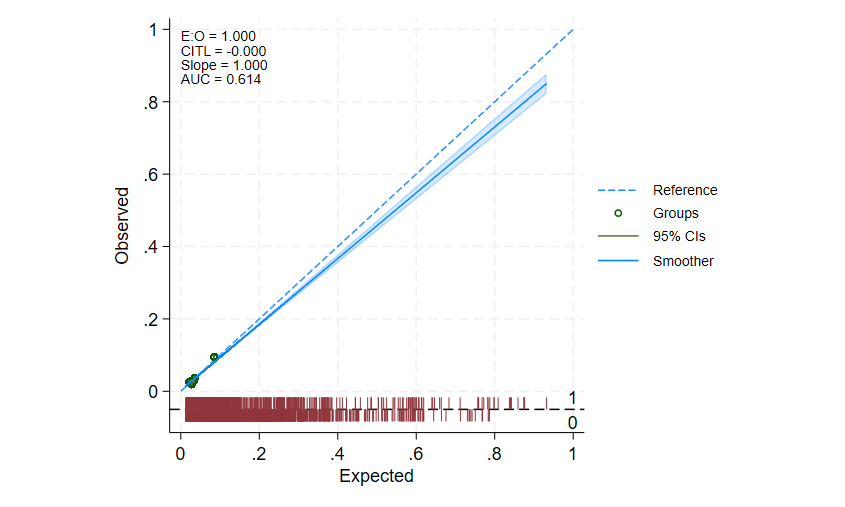


Figure S3: Bootstrap internal validation calibration plot


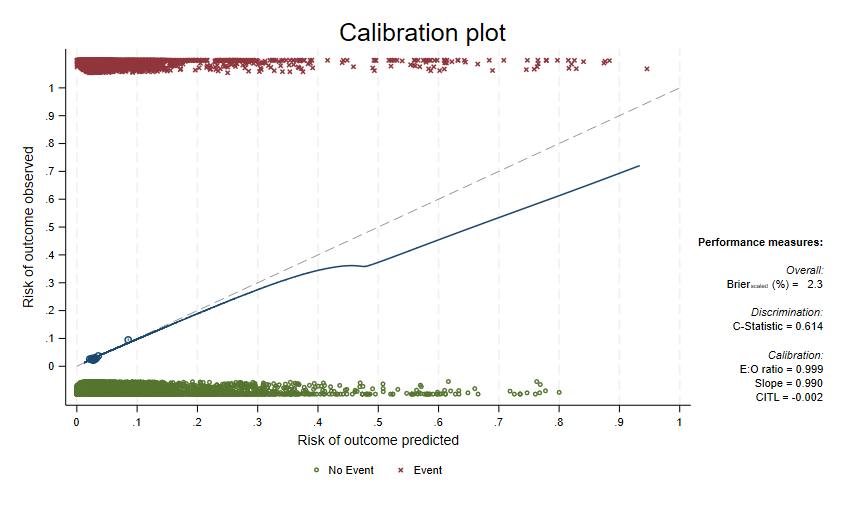

Supplement: online supplemental file 2 [file bmjpo-9-1-s002.docx]
