# Supplementary material for: Predictors of persisting symptoms after concussion in children following a traumatic brain injury: a longitudinal retrospective cohort study
Source: BMJ Paediatr Open. 2025 Apr 5;9(1):e003036. doi: 10.1136/bmjpo-2024-003036 (PMC11973773; doi:10.1136/bmjpo-2024-003036)
Supplement: Supplementary file 3 [file bmjpo-2024-003036.reviewer_comments.pdf]

PEER REVIEW HISTORY

BMJ Paediatrics Open publishes all reviews undertaken for accepted manuscripts. Reviewers are asked to complete a checklist review form and are provided with free text boxes to elaborate on their assessment. These free text comments are reproduced below.

ARTICLE DETAILS

**TITLE (PROVISIONAL)**	Predictors of Persisting Symptoms after Concussion in Children Following a Traumatic Brain Injury: a longitudinal retrospective cohort study
**AUTHORS**	Wilson, Rebecca; Jackson, Joni; Birnie, Kate; Ijaz, Sharea; Booker, Matthew; Burrell, Alex; Haythornthwaite, Giles; Hong, Jialan; Lyttle, Mark; Pocock, Lucy; Scott, Lauren; Williams, Cathy; Wright, Ingram; Savovic, Jelena; Mytton, Julie; Redaniel, Maria Theresa

VERSION 1 - REVIEW

**REVIEWER NAME**	***Rob Forsyth***
**REVIEWER AFFILIATION**	Newcastle University, Institute of Neuroscience
**REVIEWER CONFLICT OF INTEREST**	N/A
**AI DISCLOSURE**	No
**DATE REVIEW RETURNED**	25-Sep-2024

**GENERAL COMMENTS**	The issue of triaging apparently trivial, mild traumatic brain injury (TBI) in Emergency Department ED settings is an important and topical one. With concerns about early dementia emerging in the adult TBI literature, particularly in the context of high profile former professional athletes, there has been an understandable increase in research interest into concussion and Post Concussion Syndrome (PCS) in children and young people, again particularly in the context of organised sport. At this time the relevance of concussion and PCS to late dementia risk is far from clear and there is some concern that the two are being conflated. One of the widely recognised pragmatic challenges is that of identifying acute predictors of PCS.<br><br>This paper is a useful addition to the literature in confirming my (and I think other practitioners) clinical impression that the development of PCS relates more to injury-independent than injury-dependent factors i.e. premorbid risk factors than injury mechanisms per se.<br><br>The strengths of this paper are its epidemiological rigour and sample size, linking ED attendances to primary care registers. However, as the authors rightly acknowledge, the main weakness of the study is that they are using help-seeking for PCS as a proxy for PCS, together with the limitation of coding in primary care records.<br><br>In my opinion, the authors have taken an appropriately thorough and considered approach to the challenges of identifying PCS given the limitations of ICD-10 and CPRD terms. The limitations of the study are appropriately discussed. The authors identify older age as a risk factor for development of PCS and speculate as to reasons for this. An additional factor that might be considered is confounds between

	age at injury and mechanism of injury, such as injuries sustained in organised sport.<br><br>The authors are appropriately cautious about speculating as to reasons for the association of PCS with female sex. Again, informally it is recognised that PCS symptoms share many similarities with migraine and often respond to migraine treatments. The authors do identify an association with prior headache. I don't know whether their coding would allow distinction between migraines and other headache types. But it may be possible to confirm that this is a specific association between PCS and a past medical history of migraine, which is of course commoner in post-pubertal females.<br><br>Overall, I think this is a well-executed and considered study with appropriate discussion of limitations. The one factor I would have liked to have seen was incorporation of injury severity indices, such as duration of any of loss of consciousness or periods of confusion or disorientation. I strongly suspect that this was not included because of these factors being poorly documented and recorded, but it would be helpful to have discussion of this point. Otherwise, I have no additional criticisms of the paper.

**REVIEWER NAME**	***Keith Yeates***
**REVIEWER AFFILIATION**	University of Calgary, Psychology
**REVIEWER CONFLICT OF INTEREST**	N/A
**AI DISCLOSURE**	No
**DATE REVIEW RETURNED**	26-Nov-2024

**GENERAL COMMENTS**	This paper presents a retrospective, longitudinal cohort study that examines factors associated with symptoms or diagnoses of post-concussion syndrome (PCS) in a large cohort identified using administrative data from the UK. In a multivariable model, the binary outcome representing PCS was associated with older age, female sex, Asian or mixed ethnicity, and preinjury history of headache, learning disabilities, ADHD, anxiety, depression, and sleep disorder.<br><br>Although the topic is important and the results are generally consistent with existing research, the study has several shortcomings that dampen my overall enthusiasm. To start, the diagnosis of PCS itself is badly outdated, because of the lack of evidence for a well-defined syndrome, the inconsistency in diagnostic criteria across the ICD and DSM, and the high rates of false positives in non-concussed samples (i.e., the criteria are overly broad and non-specific). PCS was initially superseded by the term persistent post-concussive symptoms (PPCS) and, more recently, by persisting symptoms after concussion (PSaC; DOI: 10.1002/pmrj.12884). I suggest the authors change the title of the study and reframe the study itself accordingly.<br><br>In the Introduction, the authors suggest there is a “lack of reliable evidence” regarding the prediction of children at risk for PPCS. This is an overstatement. A large body of evidence has accrued since a key systematic review by Zemek et al in 2013 (doi: 10.1001/2013.jamapediatrics.216), including large-scale prospective studies in Canada and Australia (doi: 10.1001/jama.2016.1203; doi: 10.1136/bmjopen-2017-017012A-CAP; doi.org/10.1136/bmjopen-

	2015-009427). The collective evidence has been summarized in various sources, including the CDC guidelines for mild traumatic brain injury (doi: 10.1001/jamapediatrics.2018.2847) and a recent narrative review in Lancet Child and Adolescent Health (doi: 10.1016/S2352-4642(23)00193-1). More evidence is certainly welcome, but the available evidence is substantial and should be acknowledged.<br><br>The operational definition of PCS is potentially problematic, particularly when it rests on the report of only 1 or 2 symptoms. Studies of different methods for identifying PPCS show that methods that rely on simple symptom counts yield high rates of false positives in children without concussion and likely over-identify PPCS in children with concussion, not least because the symptoms are non-specific. I would like to know what proportion of the cohort was identified based on an ICD diagnosis versus only symptom report, and whether the prediction of PCS differed for those subgroups.<br><br>The 3-month lower limit for identifying symptoms may be overly restrictive. Most recent studies define PPCS/PSaC as symptoms lasting longer than 1 month. Symptoms occurring closer in time to the index injury are more likely to be linked to that injury. I am concerned that cases of PCS identified based on symptom reports alone much later in time post-injury may reflect pre-injury difficulties unrelated to concussion. This seems especially likely given that preinjury difficulties of various sorts were the strongest predictors of PCS. In other words, many of the symptoms used to identify PCS may have pre-existed the concussion.<br><br>I was surprised that injury severity was not considered as a potential predictor given the wide range of diagnostic codes included. Although severity can be difficult to gauge using ICD diagnoses, key distinctions are possible (e.g., between concussion and more severe injuries involving intracranial lesions). For example, algorithms have been proposed for translating ICD diagnoses to Abbreviated Injury Scale scores.<br><br>I also wondered whether the inclusion of the entire spectrum of injuries may have influenced the findings. Most research on persisting symptoms is focused on mild TBI/concussion. I am curious how many children in the cohort had diagnoses indicative of more severe injury, and whether results would be similar if analyses were restricted to those with concussion/mild TBI.<br><br>The relationship of PCS to age is unlikely to be linear given the wide range encompassed by this cohort (1-17 years). Both the youngest children and adolescents may be at risk for poorer outcomes. I wondered if non-linear relationships with age were considered in the analyses.<br><br>I found it interesting that area deprivation was a significant predictor in models measuring PCS over a longer time frame (Table S5, PCS defined on symptoms up to 3 years post-injury). Some research has suggested that environmental factors become stronger predictors of PCS over time post-injury, while the influence of the injury itself fades. In the Discussion, the statement that, “We did not find evidence for an association between socioeconomic status and PCS,” needs to be qualified, as the association was significant in a univariate analysis and in two of the sensitivity analyses using different definitions of PCS. I do not think the authors should be so

	quick to dismiss the association of social determinants of health with the outcomes of mild TBI.<br><br>Most of the effect sizes for individual predictors were relatively small, and the C statistic for the overall model was relatively poor. Other prognostic studies have yielded better prediction (e.g., Zemek 5P rule), likely because they included predictors that are not commonly available in administrative data. This should be acknowledged in the Discussion.<br><br>As the authors note, access to care and health-seeking behavior are likely significant confounds in a sample identified using administrative records. This reflects a significant selection bias, and likely accounts for the relatively low rate of PCS identified in this cohort as opposed to prospective cohorts identified at the time of injury. I wondered if some index of pre-injury health care utilization could be constructed and used as a predictor of PCS.<br><br>The study protocol indicated that bootstrapping methods would be used to test the internal validity of statistical models. But bootstrapping is not mentioned in the paper itself. Was it conducted? The protocol also indicates that subgroup analyses based on age would be conducted, but none are reported. Were they done?<br><br>The study protocol notes that consent was not required for use of the anonymized data. But this should probably be reported in the paper itself, as should approval by the relevant ethics board.

VERSION 1 – AUTHOR RESPONSE

Reviewer 1

Comment	Response
This paper is a useful addition to the literature in confirming my (and I think other practitioners) clinical impression that the development of PCS relates more to injury-independent than injury-dependent factors i.e. premorbid risk factors than injury mechanisms per se.	Thank you very much for the kind words.
In my opinion, the authors have taken an appropriately thorough and considered approach to the challenges of identifying PCS given the limitations of ICD-10 and CPRD terms. The limitations of the study are appropriately discussed. The authors identify older age as a risk factor for development of PCS and speculate as to reasons for this. An additional factor that might be considered is confounds between age at injury and mechanism of injury, such as injuries sustained in organised sport.	Unfortunately mechanism of injury is not captured in primary care/A&E/admissions data, this would have been a valuable measure and is a limitation of our study. (This is mentioned in the Strengths and Limitations section, page 14)
The authors are appropriately cautious about speculating as to reasons for the association of	The codes included two (out of 101 codes in the code list for headaches) codes for migraine in

PCS with female sex. Again, informally it is recognised that PCS symptoms share many similarities with migraine and often respond to migraine treatments. The authors do identify an association with prior headache. I don't know whether their coding would allow distinction between migraines and other headache types. But it may be possible to confirm that this is a specific association between PCS and a past medical history of migraine, which is of course commoner in post-pubertal females.	primary care and six (out of 15) codes from A&E/admissions data, however the number of observations with these codes were so small (probably due to the sample being children and majority under 5 years) that we do not think it would be possible to investigate a meaningful or robust association between migraine and PCS.
Overall, I think this is a well-executed and considered study with appropriate discussion of limitations. The one factor I would have liked to have seen was incorporation of injury severity indices, such as duration of any of loss of consciousness or periods of confusion or disorientation. I strongly suspect that this was not included because of these factors being poorly documented and recorded, but it would be helpful to have discussion of this point. Otherwise, I have no additional criticisms of the paper.	Thank you. Yes, you are correct. Unfortunately, the dataset, which comprised only consultation codes, does not include any severity indices. We agree that this would have been a helpful addition to our study, but it remains a limitation. This has been added to the Strengths and Limitations section (page 14).

Reviewer 2

Comment	Response
Although the topic is important and the results are generally consistent with existing research, the study has several shortcomings that dampen my overall enthusiasm. To start, the diagnosis of PCS itself is badly outdated, because of the lack of evidence for a well-defined syndrome, the inconsistency in diagnostic criteria across the ICD and DSM, and the high rates of false positives in non-concussed samples (i.e., the criteria are overly broad and non-specific). PCS was initially superseded by the term persistent post-concussive symptoms (PPCS) and, more recently, by persisting symptoms after concussion (PSaC; DOI: 10.1002/pmrj.12884). I suggest the authors change the title of the study and reframe the study itself accordingly.	Thank you for bringing this to our attention, we have now defined PSaC in the Introduction (page 4) and refer to PSaC, rather than PCS, throughout.
In the Introduction, the authors suggest there is a “lack of reliable evidence” regarding the prediction of children at risk for PPCS. This is an overstatement. A large body of evidence has accrued since a key systematic review by Zemek	Thank you for raising this important point and for the suggested references. We apologise as this should have worded “lack of consistent evidence”, as we do say in the above paragraph, “Research into potential predictors of PCS is growing but is

et al in 2013 (doi: 10.1001/2013.jamapediatrics.216), including large-scale prospective studies in Canada and Australia (doi: 10.1001/jama.2016.1203; doi: 10.1136/bmjopen-2017-017012A-CAP; doi.org/10.1136/bmjopen-2015-009427). The collective evidence has been summarized in various sources, including the CDC guidelines for mild traumatic brain injury (doi: 10.1001/jamapediatrics.2018.2847) and a recent narrative review in Lancet Child and Adolescent Health (doi: 10.1016/S2352-4642(23)00193-1). More evidence is certainly welcome, but the available evidence is substantial and should be acknowledged.	inconsistent". We have added to the Introduction (page 4) the results of the Zemek 2016 paper and cited the two protocols (Bressan and Yeates). The Beauchamp review has also been referred to.
The operational definition of PCS is potentially problematic, particularly when it rests on the report of only 1 or 2 symptoms. Studies of different methods for identifying PPCS show that methods that rely on simple symptom counts yield high rates of false positives in children without concussion and likely over-identify PPCS in children with concussion, not least because the symptoms are non-specific. I would like to know what proportion of the cohort was identified based on an ICD diagnosis versus only symptom report, and whether the prediction of PCS differed for those subgroups.	The number of PCS diagnosis codes (this is across ICD and CPRD) in the cohort was very small (N=199/137,279, 0.14%). This was why we used symptoms as a way of including 'probable' PCS, although we appreciate this may include some false positives. However, the use of codes to identify probable PCS symptoms still yielded fairly low numbers (the primary outcome measure = 3.4%), much lower than other reported estimates of PCS. The use of medical attendance codes to try and capture PCS is described in the Strengths and Limitations on page 14.
The 3-month lower limit for identifying symptoms may be overly restrictive. Most recent studies define PPCS/PSaC as symptoms lasting longer than 1 month. Symptoms occurring closer in time to the index injury are more likely to be linked to that injury. I am concerned that cases of PCS identified based on symptom reports alone much later in time post-injury may reflect pre-injury difficulties unrelated to concussion. This seems especially likely given that preinjury difficulties of various sorts were the strongest predictors of PCS. In other words, many of the symptoms used to identify PCS may have pre-existed the concussion.	This is an important question and is one we considered carefully when deciding how we might try to capture probable PCS in clinical codes. As our codes are indicators of healthcare seeking and clinical diagnosis there is a lag between onset of symptom(s) and accessing healthcare and receiving a diagnosis. Particularly when some of the PCS symptoms may be thought of as less urgently requiring medical attention, compared with TBI. Many definitions of PCS state that symptoms persist for at least one month after injury, therefor our use of clinical codes had to exceed this length of time.<br><br>Further, we chose to use a three month cut-off based on previous evidence. Barlow et al (2015) and Babcock et al (2013) employed a timeframe of three months post injury. We refer to this in the Methods, under Outcomes on page 6.<br><br>Whilst pre-injury clinical factors were strong and consistent predictors of PCS, we excluded the symptoms also used in the list of PCS symptoms

	in sensitivity analysis, as a way of ensuring that we were just measuring an association between pre-injury difficulties and PCS, and we found no difference in the results.
I was surprised that injury severity was not considered as a potential predictor given the wide range of diagnostic codes included. Although severity can be difficult to gauge using ICD diagnoses, key distinctions are possible (e.g., between concussion and more severe injuries involving intracranial lesions). For example, algorithms have been proposed for translating ICD diagnoses to Abbreviated Injury Scale scores…<br><br>I also wondered whether the inclusion of the entire spectrum of injuries may have influenced the findings. Most research on persisting symptoms is focused on mild TBI/concussion. I am curious how many children in the cohort had diagnoses indicative of more severe injury, and whether results would be similar if analyses were restricted to those with concussion/mild TBI.	Unfortunately, injury severity was not available in our data. Although this may be possible using algorithms for ICD codes, the majority of our cases came from CPRD data.<br><br>We acknowledge that the experiences and outcomes for children with mild and severe TBI will be very different, however we decided to include the entire spectrum of injuries as previous research reported that the majority of cases of TBI in children are mild and these children still experiences adverse outcomes following their injury (Sariaslan et al, 2016, doi: 10.1371/journal.pmed.1002103.)<br><br>Further, as mentioned above injury severity is not routinely coded in primary care data (CPRD) we would have had to use a proxy measure of severity which would have imposed subjective judgments which could, in themselves, be criticised. The only available option for our data would have been to categorise TBIs as structural or non-structural, which would not necessarily correlate with severity of TBI either at the time of injury or discharge. We acknowledge that this is a limitation of the study and have added a couple of sentences to the Strengths and Limitations section, page 14.
The relationship of PCS to age is unlikely to be linear given the wide range encompassed by this cohort (1-17 years). Both the youngest children and adolescents may be at risk for poorer outcomes. I wondered if non-linear relationships with age were considered in the analyses.	Thank you for this interesting question. The age of the study participants was heavily skewed towards younger age, 50% of participants were below 4.75 years old. For this reason we decided not to categorise age as the numbers in the older age groups were small. We have added an additional sensitivity analysis stratifying the primary analysis by age (<5 years old and ≥5 years), in Supplementary Tables S6. These analyses do confirm that, when stratifying by age group, the youngest children and the oldest children have the largest likelihood of PCS. We discuss these results at the end of the Results section (page 10). The age-adjusted associations are mostly similar to the primary analysis with a few exceptions that have been added to the Discussion.

I found it interesting that area deprivation was a significant predictor in models measuring PCS over a longer time frame (Table S5, PCS defined on symptoms up to 3 years post-injury). Some research has suggested that environmental factors become stronger predictors of PCS over time post-injury, while the influence of the injury itself fades. In the Discussion, the statement that, “We did not find evidence for an association between socioeconomic status and PCS,” needs to be qualified, as the association was significant in a univariate analysis and in two of the sensitivity analyses using different definitions of PCS. I do not think the authors should be so quick to dismiss the association of social determinants of health with the outcomes of mild TBI.	Thank you for highlighting this discussion point. We have amended the statement about not finding evidence of an association, and have added the following to the Discussion (page 12): “However, the two sensitivity analyses that included longer follow-up periods for suspected PSaC (up to 3 years) did show associations between the most deprived quintiles and increased risk of PSaC, compared with the least deprived. This may support the hypothesis that social and environment factors have a larger impact on more long-term recovery following TBI.”
Most of the effect sizes for individual predictors were relatively small, and the C statistic for the overall model was relatively poor. Other prognostic studies have yielded better prediction (e.g., Zemek 5P rule), likely because they included predictors that are not commonly available in administrative data. This should be acknowledged in the Discussion.	We have added the following statement to the Discussion (page 14): “The C-statistic for the full regression model was lower than that reported previously by Zemek et al. There are several possible reasons for this; Zemek and colleagues' model was improved by using predictors identified both using physicians' judgement in addition to those from the derived prediction model, and they utilised variables generally not available in routinely collected data, whereas our study was likely limited by the available data in linked CPRD datasets.”
As the authors note, access to care and health-seeking behavior are likely significant confounds in a sample identified using administrative records. This reflects a significant selection bias, and likely accounts for the relatively low rate of PCS identified in this cohort as opposed to prospective cohorts identified at the time of injury. I wondered if some index of pre-injury health care utilization could be constructed and used as a predictor of PCS.	We agree that using a measure healthcare seeking for PCS or suspected PCS is likely to lead to an underestimation of PCS.<br><br>In another study (currently unpublished) exploring the factors associated with TBI in adolescents, we included measures of number of GP visits, A&E visits and hospitalisations, as we hypothesised that these adolescents could either be (a) higher users of healthcare or (b) more injury-prone. We did not find associations between these measures of healthcare access and TBI in adolescents. For this reason we decided not to include these variables when assessing PCS in the present study, plus there seemed less of a rationale for doing so in a cohort with a medically attended TBI.
The study protocol indicated that bootstrapping methods would be used to test the internal validity of statistical models. But bootstrapping is not mentioned in the paper itself. Was it conducted? The protocol also indicates that subgroup	We have used bootstrapping methods for internal validation which provided no real evidence of model over-fitting (the C-statistic using bootstrapping was 0.615, compared with 0.616 from the full model. Text has been added to the

analyses based on age would be conducted, but none are reported. Were they done?	Methods (page 7) and the Results (page 9) and the calibration plot to the Supplementary Materials (Figure S3).<br><br>We have added an additional sensitivity analysis stratifying the primary analysis by a binary measure of age (<5 years old and ≥5 years), in Supplementary Tables S6.
The study protocol notes that consent was not required for use of the anonymized data. But this should probably be reported in the paper itself, as should approval by the relevant ethics board.	We have added the following statement to the manuscript (page 5): "Ethical approval for the use of CPRD's anonymised data is covered by CPRD's existing ethical approval from the National Research Ethics Service Committee (NRES)."

VERSION 2 – REVIEW

**REVIEWER NAME**	***Keith Yeates***
**REVIEWER AFFILIATION**	University of Calgary, Psychology
**REVIEWER CONFLICT OF INTEREST**	N/A
**AI DISCLOSURE**	No
**DATE REVIEW RETURNED**	12-Feb-2025

**GENERAL COMMENTS**	Overall, the authors have been very responsive to the previous reviews, and the paper is substantially improved. I have one minor remaining concern. In the Discussion, the authors state, "Zemek and colleagues' model was improved by using predictors identified using physicians' judgement in addition to those from the derived prediction model." This has things turned around. No predictors were identified on the basis of physician judgment, which was measured by asking on treating physicians a single question: "How likely is this patient to develop persistent symptoms beyond 1 month?" A more accurate statement would be, "Zemek and colleagues' model combined predictors from the derived prediction model with physician judgment regarding the likelihood of recovery."

VERSION 2 – AUTHOR RESPONSE

Reviewer 2

Comment	Response
A more accurate statement would be, "Zemek and colleagues' model combined predictors from the derived prediction model with physician judgment regarding the likelihood of recovery."	Thank you for highlighting this error, we have added these words to the top paragraph on page 14, "There are several possible reasons for this; Zemek and colleagues' model combined predictors from the derived prediction model with physician judgment regarding the likelihood of recovery, and they utilised variables generally not

	available in routinely collected data, whereas our study was likely limited by the available data in linked CPRD datasets."
